# Effect of Seven-Valent Pneumococcal Conjugate Vaccine on *Staphylococcus aureus* Colonisation in a Randomised Controlled Trial

**DOI:** 10.1371/journal.pone.0020229

**Published:** 2011-06-10

**Authors:** Elske J. M. van Gils, Eelko Hak, Reinier H. Veenhoven, Gerwin D. Rodenburg, Debby Bogaert, Jacob P. Bruin, Loek van Alphen, Elisabeth A. M. Sanders

**Affiliations:** 1 Department of Pediatric Immunology and Infectious Diseases, Wilhelmina Children's Hospital, University Medical Center Utrecht, Utrecht, the Netherlands; 2 Research Center Linnaeus Institute, Spaarne Hospital Hoofddorp, Hoofddorp, the Netherlands; 3 Department of PharmocoEpidemiology and PharmacoEconomics, University Medical Center Groningen, Groningen, the Netherlands; 4 Department of Epidemiology, University Medical Center Groningen, Groningen, the Netherlands; 5 Regional Public Health Laboratory of Kennemerland, Haarlem, the Netherlands; 6 Netherlands Vaccine Institute, Bilthoven, the Netherlands; Instituto Butantan, Brazil

## Abstract

**Background:**

Heptavalent pneumococcal conjugate vaccine (PCV7) shifts nasopharyngeal colonisation with vaccine serotype pneumococci towards nonvaccine serotypes. Because of the reported negative association of vaccine serotype pneumococci and *Staphylococcus aureus* in the nasopharynx, we explored the effect of PCV7 on nasopharyngeal colonisation with *S. aureus* in children and parents.

**Methodology/Principal Findings:**

This study was part of a randomised controlled trial on the effect of PCV7 on pneumococcal carriage, enrolling healthy newborns who were randomly assigned (1∶1∶1) to receive PCV7 (1) at 2 and 4 months of age (2) at 2, 4 and 11 months or (3) no PCV7 (controls). Nasopharyngeal colonisation of *S. aureus* was a planned secondary outcome. Nasopharyngeal swabs were obtained from all children over a 2-year period with 6-months interval and from one parent at the child's age of 12 and 24 months and cultured for *Streptococcus pneumoniae* and *S. aureus*. Between July 2005 and February 2006, 1005 children were enrolled and received either 2-doses of PCV7 (n = 336), 2+1-doses (336) or no dose (n = 333) before PCV7 implementation in the Dutch national immunization program. *S. aureus* colonisation had doubled in children in the 2+1-dose group at 12 months of age compared with unvaccinated controls (10.1% versus 5.0%; p = 0.019). A negative association for co-colonisation of *S. pneumoniae* and *S. aureus* was observed for both vaccine serotype (adjusted odds ratio (aOR) 0.53, 95% confidence interval (CI) 0.38–0.74) and nonvaccine serotype pneumococci (aOR 0.67, 95% CI 0.52–0.88).

**Conclusions/Significance:**

PCV7 induces a temporary increase in *S. aureus* colonisation in children around 12 months of age after a 2+1-dose PCV7 schedule. The potential clinical consequences are unknown and monitoring is warranted.

**Trial Registration:**

ClinicalTrials.gov NCT00189020

## Introduction

Seven-valent CRM197-conjugated pneumococcal vaccine (PCV7) provides protection against vaccine serotype invasive pneumococcal disease (IPD) [1], pneumonia and acute otitis media in young children [2]. In the nasopharynx, PCV7 reduces colonisation with vaccine serotype pneumococci [3]–[5] and due to herd effects nasopharyngeal carriage of pneumococcal vaccine serotypes in children has virtually disappeared several years after widespread infant PCV7 vaccination [6]. The vacant ecological niche in the nasopharynx seems immediately occupied by nonvaccine serotype pneumococci resulting in a shift in serotype carriage with limited to no net reduction in overall pneumococcal carriage [3], [4], [5], [7].

*S. pneumoniae* is a frequent coloniser in the dynamic nasopharyngeal *milieu interieure* and pneumococcal serotype shifts following PCV7 vaccination may affect other bacteria present in the nasopharynx. For example, several studies found a negative association between the presence of *S. aureus* and PCV7 vaccine serotype pneumococci in the nasopharynx in unvaccinated children [8]–[10]. This may imply an upcoming presence of *S. aureus* after PCV7-vaccinations. A higher staphylococcal nasal load has been suggested to lead to higher dispersal to other extra-nasal sites harbouring *S. aureus* like the nasopharynx. Furthermore, *S. aureus* colonisation is associated with a higher risk of infection [11]. *S. aureus* is an important cause of respiratory tract infections such as chronic recurrent otitis media and pneumonia, skin infections and community-acquired bloodstream infections in particular in children under 5 years of age, with increasing prevalence of multi resistant strains [12].

Investigating the effect of PCV7 on nasopharyngeal presence of bacteria that may behave as disease pathogens is relevant. As part of a longitudinal randomised controlled trial on the effects of a 2-dose and 2+1-dose PCV7-schedule on nasopharyngeal pneumococcal colonisation during the first two years of life, we assessed the effects of PCV7 on *S. aureus c*olonisation in children and parents as secondary outcomes.

## Methods

The protocol for this trial and supporting CONSORT checklist are available as supporting information; see Checklist S1, Protocol S1 and Summary Protocol S1.

### Participants and Ethics Statement

This was a randomized controlled study including healthy newborns from the western part of The Netherlands. The trial methodology and results for pneumococcal carriage efficacy have been published previously [5], [13]. Written informed consent was obtained from both parents or guardian. This study received approval by an acknowledged national ethics committee (Stichting Therapeutische Evaluatie Geneesmiddelen) from the Netherlands and was undertaken in accordance with the European Statements for Good Clinical Practice, which includes the provisions of the Declaration of Helsinki. Participants did not receive any financial compensation.

### Randomization and Blinding

Infants were randomly allocated (1∶1∶1) to receive (1) PCV7 at the age of 2 and 4 months (2-dose group); (2) PCV7 at 2, 4, and 11 months (2+1-dose group); or (3) no PCV7 (unvaccinated control group). Randomisation was performed during the first home-visit via a computer-randomisation interface. The assigned vaccination schedule was communicated with the well baby clinic where vaccinations were administered. The study nurses and parents were unmasked to the intervention assignment but the outcome assessors were blinded throughout the study and the randomisation key was not disclosed until after the study.

### Procedures

Deep nasopharyngeal swabs were taken transnasally by trained study nurses according to WHO standard procedures [14] during home visits at age 6 weeks and at age 6, 12, 18 and 24 months and from parents at the child's age of 12 and 24 months. From parents, also a transoral nasopharyngeal swab was collected, as the pneumococcal yield is known to be higher in adults when taking both swabs [15]. Transoral swabs were taken under direct observation of the posterior pharynx with a rigid cotton-wool swab. After sampling, swabs were immediately inoculated in Transwab (modified Amies) transport medium and stored at room temperature. Swabs were plated within 24 hours onto two 5% sheep blood agar plates, 1 with 5 mg/L gentamicin and 1 without. Agar plates were incubated at 35°C for 48 hours (the gentamicin plate with raised CO_2_). Isolates were identified using colony morphology and conventional methods of determination. Serotyping of *S. pneumoniae* was performed by capsular swelling method (Quellung reaction) using type-specific antisera from the Statens Seruminstitut (Copenhagen, Denmark). A questionnaire on risk factors for nasopharyngeal bacterial colonisation was obtained with each nasopharyngeal swab.

### Sample Size and Statistical Methods

Sample size calculation for the trial was based on the main outcome measure of the study, vaccine serotype pneumococcal carriage in the second year of life, resulting in a sample size of 330 infants per group including a 10% dropout rate as previously described [5]. The present analyses were planned as secondary outcomes. All statistical analyses in the trial were carried out according to the intention-to-treat principle, meaning that all available data from all randomized participants were analyzed according to the assigned intervention. Parents of twins were excluded from the analyses. Because the dropout rate (<2%) and the amount of missing data (<2%) were low, available data were analyzed without using imputation methods. We used a repeated measurements model taking more than one measurement per child into account using generalized linear models procedure in SPSS version 17.0 (SPSS Inc, Chicago, Illinois) with *S. aureus* colonisation as the dependent variable and age as within-subject variable with an unstructured correlation structure [16]. Next to the assigned vaccination schedule (no PCV7, 2-dose, or 2+1-dose), potential risk factors for *S. aureus* colonisation as identified in recent studies were included in the model: day care attendance (no/yes), presence of siblings (no/yes), sex (male/female), presence of *S. pneumoniae* (no carrier, vaccine type, or nonvaccine type carrier) [9], [10]. Data from all infants from age 6 months up to 24 months were included in the model. Furthermore, a multivariate logistic regression model was constructed for each sampling moment with *S. aureus* colonisation as the dependent variable and aforementioned covariates to explore possible changes over time. P-values smaller than 0.05 were considered significant and all reported p-values are 2-sided. The trial is registered with the ClinicalTrials.gov, number NCT00189020.

## Results

As reported previously [5], [13], 1005 infants were enrolled between July 7, 2005 and February 9, 2006, well before the introduction of PCV7 in the Dutch national immunization program for infants who were born after March 31 2006 in a 3+1-dose schedule without catch-up campaign [17]. In the control group, 333 children in were enrolled and 336 children in both the 2-dose and 2+1-dose group. Two children were excluded after randomisation because one of the parental approval signatures could not be obtained. The study ended when the last child had reached 24 months of age at February 14, 2008. There were no major differences in demographics or distribution of risk factors (e.g. number of siblings, day care attendance) between the 3 study groups [5].

### *S. aureus* colonisation in unvaccinated control children and their parents

**Figure 1:**
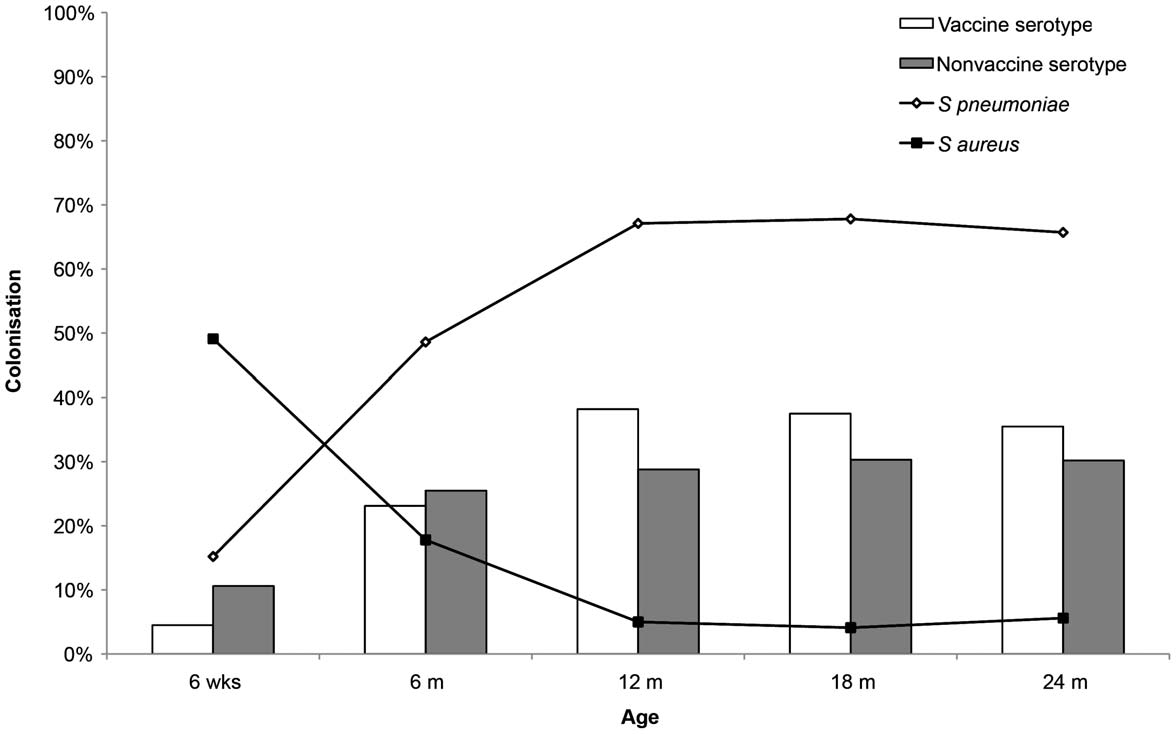
*S. pneumoniae* and *S aureus* colonisation in PCV7-unvaccinated control children in The Netherlands. PCV7 indicates 7-valent pneumococcal conjugate vaccine; wks – weeks; m – months.

In unvaccinated children in the control group, *S. aureus* colonisation was 49.1% at 6 weeks (95% confidence interval (CI), 43.7%–54.5%) and declined towards 5.0% (95% CI, 3.1%–8.0%) from 12 months and onwards. As reported previously, pneumococcal carriage increased from 15.2% (95% CI 11.7%–19.4%) at age 6 weeks to 67.1% (95% CI 61.8%–72.0%) at age 12 months and remained stable hereafter [5]. (Figure 1) In parents of unvaccinated control children, the colonisation rate for *S. aureus* at the child's age of 12 months was 28.9% (95% CI 24.1%–34.2%) and 16.9% (95% CI 13.1%–21.6%) at the child's age of 24 months.

### Effect of PCV7 on *S. aureus* colonisation

**Figure 2:**
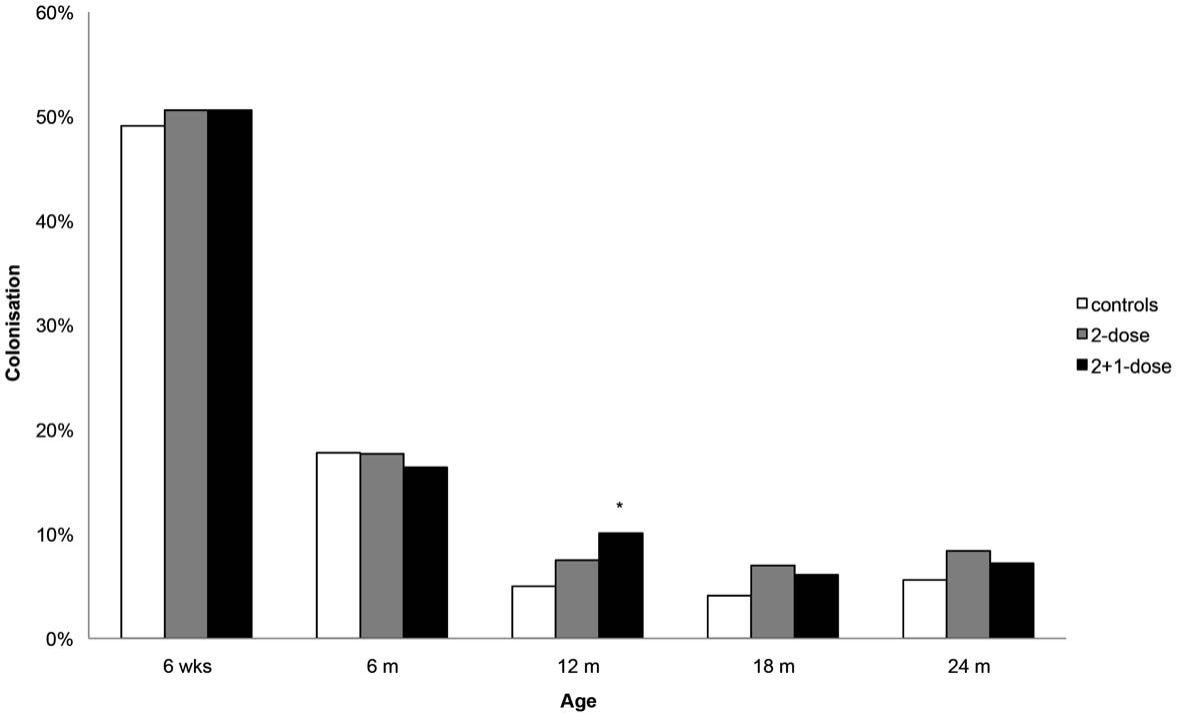
Effect of PCV7 on nasopharyngeal *S aureus* colonisation in children. PCV7 indicates 7-valent pneumococcal conjugate vaccine; wks – weeks; m – months * P<0.05 versus control group using Generalised Estimating Equations-model.

A trend toward higher colonisation of *S. aureus* after PCV7 vaccination in the first 2 years of life was observed, though not significant overall (overall significance test of model effects: p = 0.097). However, when analyzing different time points to investigate temporary effects of the different vaccination schedules, *S. aureus* carriage was found doubled at 12 months in children in the 2+1-dose group, 1 month after receiving the booster dose (10.1%, 95% CI 7.4%–13.8%) compared to controls (5.0%, 95% CI 3.1%–8.0%; p = 0.019).(Figure 2) The point estimate for *S. aureus* nasopharyngeal presence in children in the 2-dose group without the booster at 11 months was in between controls and the group that had received the booster dose (7.5%, 95% CI 5.1%–10.8%). At 18 and 24 months, point estimates for *S. aureus* carriage were still higher in vaccinated children of both vaccine groups compared to controls; however with low carriage rates these differences were no longer significant. In parents, point estimates for nasopharyngeal presence of *S. aureus* did not significantly differ between the three groups at the child's age of 12 and 24 months, although the point estimate in parents of the 2+1-dose group (25.5%, 95% CI 21.1%–30.6%) was higher than in parents of children in the 2-dose group (20.4%, 95% CI 16.3%–25.2%) and control group (20.3%, 95% CI 16.1%–25.2%) at the child's age of 24 months. In all three groups, point estimates for *S. aureus* colonisation at the child's age of 24 months were lower compared to at age 12 months. (Table 1)

**Table 1:** Nasopharyngeal colonisation of *S. pneumoniae* and *S. aureus* in parents of unvaccinated children and of children receiving a 2-dose or 2+1-dose PCV7-schedule.

	Parents of unvaccinated control children			Parents of children with a 2-dose PCV7-schedule			Parents of children with a 2+1-dose PCV7-schedule		
	n	%	95% CI	n	%	95% CI	n	%	95% CI
***S. pneumoniae***									
Age 12 mo^a^	84	27.5%	22.7%–32.7%	74	23.3%	18.9%–28.1%	75	22.9%	18.6%–27.6%
Age 24 mo^b^	50	16.9%	12.9%–21.5%	63	20.4%	16.2%–25.2%	71	22.1%	17.8%–26.9%
***S. aureus***									
Age 12 mo^a^	88	28.9%	24.0%–34.1%	91	28.6%	23.8%–33.8%	90	27.4%	22.8%–32.5%
Age 24 mo^b^	60	20.3%	16.0%–25.1%	63	20.4%	16.2%–25.2%	82	25.5%	21.0%–30.5%

Parents of twins (n = 15 pairs) were excluded. Analyses were performed with Chi-square or 2-sided Fisher Exact test where appropriate.

a for the control group: n = 305; for the 2-dose group: n = 318; for the 2+1-dose group: n = 328.

b for the control group: n = 296; for the 2-dose group: n = 309; for the 2+1-dose group: n = 321.

### Co-colonisation for *S. aureus* and *S. pneumoniae*

*S. aureus* colonisation was higher in non-pneumococcal carriers compared to pneumococcal carriers at all sampling moments. Analyzing the overall presence of co-colonisation for *S. aureus* and *S. pneumoniae* during follow-up between 6 months and 24 months of age, a negative association for both vaccine serotype (aOR 0.53, 95% CI 0.38–0.74) and to a lesser extent nonvaccine serotype pneumococci (aOR 0.67, 95% CI 0.51–0.88) was noted. Furthermore, day care attendance and siblings in the household and increasing age were all negatively associated with *S. aureus* colonisation.(Table 2) In multivariate logistic regression models for each different sampling moment at different ages and adjusting for PCV7 vaccination schedule, day care attendance, presence of siblings and sex, colonisation with vaccine serotype pneumococci and nonvaccine serotype pneumococci in general appeared negatively associated with *S. aureus* presence though point estimates varied slightly in time.(Table 3) In parents, no significant negative associations between *S. pneumoniae* and *S. aureus* were observed. At the child's age of 12 months, *S. aureus* colonisation in parents carrying pneumococci was 23.4% (95% CI 18.4%–29.2%) and in non-pneumococcal carriers 29.8% (95% CI 26.5%–33.2%). At the child's age of 24 months, *S. aureus* colonisation was 20.7% (95% CI 15.4%–27.1%) in pneumococcal carriers and 22.5% (95% CI 19.6%–24.6%) in non-pneumococcal carriers.

**Table 2:** Risk factors for *S. aureus* colonisation in children between 6 and 24 months of age using a Generalized Estimating Equations-model.

	Adjusted OR^a^	95% CI
Vaccination schedule		
No PCV7	Reference	
2-dose	1.04	0.69–1.57
2+1-dose	0.90	0.60–1.37
Age		
6 months	Reference	
12 months	**0.27**	**0.15–0.48**
18 months	**0.22**	**0.12–0.41**
24 months	**0.32**	**0.19–0.55**
Day care attendance	**0.53**	**0.41–0.69**
Siblings present in household	**0.75**	**0.57–0.97**
Female sex	0.86	0.67–1.10
*S. pneumoniae* colonisation		
No carrier	Reference	
Vaccine type carrier	**0.53**	**0.38–0.74**
Nonvaccine type carrier	**0.67**	**0.52–0.88**

OR – Odds ratio; CI – Confidence interval; PCV7 – 7-valent pneumococcal conjugate vaccine.

a Calculated using a Generalized Estimating Equations model with *S. aureus* colonisation as dependent variable and age as within-subject variable with un unstructured correlation structure. Data from all infants at age 6, 12, 18 and 24 months were included in the model; data from age 6 weeks were excluded since day care attendance was not present at this age.

**Table 3:** Distribution and odds ratios of colonisation with *S. aureus* adjusted for co-colonisation with *S. pneumoniae* and other risk factors.

	*S. pneumoniae* negative		*S. pneumoniae* positive					
			Nonvaccine types			Vaccine types		
*S. aureus* positive	n/N	%	n/N	%	aOR (95% CI)^a^	n/N	%	aOR (95% CI)^a^
**6 weeks** a	435/829	52.5%	43/115	37.4%	**0.58 (0.38–0.88)**	24/58	41.4%	0.68 (0.39–1.19)
**6 months** b	108/493	21.9%	40/285	14.0%	**0.62 (0.41–0.94)**	23/213	10.8%	**0.47 (0.28–0.77)**
**12 months** b	41/373	11.0%	20/343	5.8%	0.68 (0.38–1.22)	14/271	5.2%	0.70 (0.36–1.37)
**18 months** b	33/365	9.0%	16/359	4.5%	0.55 (0.29–1.05)	7/249	2.8%	**0.36 (0.15–0.86)**
**24 months** b	42/403	10.4%	21/373	5.6%	0.72 (0.40–1.27)	7/210	3.3%	**0.43 (0.18–0.99)**

a Calculated using a multivariate logistic regression model also including PCV7 vaccination schedule (none, 2-dose, 2+1-dose PCV7 schedule), presence of siblings in the household (no/yes), and sex (male/female).

b Calculated using a multivariate logistic regression model also including PCV7 vaccination schedule (none, 2-dose, 2+1-dose PCV7 schedule), day care attendance (no/yes), presence of siblings in the household (no/yes), and sex (male/female).

## Discussion

We describe for the first time a randomized, controlled longitudinal trial exploring the effect of PCV7 on nasopharyngeal *S. aureus* colonisation. We found, even after a reduced primary dose PCV7-schedule with 2 instead of 3 primary vaccinations before the age of 6 months, a temporary but distinct 2-fold increase in *S. aureus* nasopharyngeal colonisation in PCV7 vaccinated infants at 12 months of age shortly after receiving a booster dose. This means that for every 20 children vaccinated with PCV7, 1 extra child is colonised with *S. aureus* in the nasopharynx. Also a negative association for both vaccine serotype pneumococci and to a lesser extent nonvaccine serotype pneumococci and *S. aureus* was observed in children but not in parents. Increased *S. aureus* colonisation at a vulnerable age may have clinical consequences.

The observed temporary increase in *S. aureus* coincides with the previously reported strong decline in vaccine serotype pneumococcal carriage at the age of 12 months, one month after the administration of the booster dose [5]. Two clinical studies, one in otitis media patients and one in primary care visiting children, did not observe an effect of PCV7 on *S. aureus* nasopharyngeal colonisation [18], [19]. However, these were both cross-sectional studies in children of varying age (6 months to 7 years) and thus a temporary effect related to the vaccination scheme may have easily been missed [20]. Furthermore, these studies were performed in a PCV7 vaccinated population with established herd effects which means less dynamic changes after vaccination since circulation and carriage of vaccine serotypes was already low. No effect of a 3-primary dose schedule with 9-valent pneumococcal conjugate vaccine in infancy on *S. aureus* colonisation was observed in a colonisation study in children that participated in a randomized, controlled trial [21]. However, this concerned a long-term follow-up study in children at the age of 5 years. We observed only a temporary significant effect particularly around the child's first birthday and shortly after the booster vaccination when the largest changes in carriage from vaccine to nonvaccine serotypes occurred, although point estimates for *S. aureus* were higher in vaccinated children compared to unvaccinated children throughout the second year of life. The fact that the increase in *S. aureus* colonisation was no longer significant at 18 months or later may well be due to underpowering of the study with respect to *S. aureus* colonisation rates, since these are much lower compared to S. *pneumoniae* rates in the second year of life.

The demonstrated negative association between *S. pneumoniae*, in particular vaccine serotypes and *S. aureus* is in line with several previous ecological studies that reported such a negative association [8]–[10]. However, we now also found a negative association for nonvaccine serotype pneumococci and *S. aureus*, albeit less strong, in contrast to a previous Dutch study in 3097 unvaccinated children aged 1 to 19 years before PCV7 implementation in the national immunisation program in the Netherlands [8]. This may be due to differences between both studies in the nonvaccine serotype pool, either due to age differences or as a result of pneumococcal serotype replacement in our study. In our study, we saw no association between presence of *S. aureus* and pneumococci in parents, which is in line with a previous study in adults [9]. *S. aureus* colonisation dynamics in children are different from adults, with frequent intermittent colonisation with different strains in children as opposed to persistent colonisation in adults [22]. Next to bacterial potential interference between *S. pneumoniae* and *S. aureus* in infants [23]–[25], the presence of other bacteria that together compose the nasopharyngeal microbiome in adults differs from in children and this may affect interactions. Also, the adult anatomy of the nose may influence interactions between *S. aureus* that has its primary niche in the anterior nares and *S. pneumoniae* in the nasopharynx. Last, the influence of mature immunity in adults may affect this interaction. For instance, in HIV-1-positive hospitalized children, no association between *S. aureus* and *S. pneumoniae* was observed [10].

Day care attendance, presence of siblings in the household and increasing age, all well-known risk factors for *S. pneumoniae* acquisition, were negatively associated with *S. aureus* colonisation. A recent study by Regev-Yochay et al. suggested that the inverse relation between pneumococci and *S. aureus* might not be associated with the pneumococcal capsule per se and that other characteristics such as pneumococcal pilus formation may play a role [26]. Also, in vitro and in vivo studies have demonstrated that the interference between the 2 pathogens may be related to hydrogen peroxide production by *S. pneumoniae*, which is bactericidal to *S. aureus* [23], [24], through lethal induction of resident prophages and subsequent lysogeny [25]. Furthermore, we need to be aware that *S. pneumoniae* is just one of many inhabitants of the dynamic nasopharyngeal niche and interactions may be more complex and may involve other bacteria and/or viruses as well. Understanding the underlying pathophysiological mechanism for the observed interaction requires further investigation of the nasopharyngeal microbiome.

The consequences of the observed temporary increase in *S. aureus* colonisation need further evaluation since an increased risk of (nosocomial) *S. aureus* infection has been shown in *S. aureus* carriers [11]. Also, a previous randomized controlled study in children with a history of recurrent otitis media by our group showed an increase of *S. aureus* in middle-ear fluids of children with otitis after receiving pneumococcal vaccinations [27]. In this study, PCV7 conjugate vaccinations followed by a 23-valent polysaccharide booster vaccination induced a firm decline in PCV7 serotype nasopharyngeal carriage and replacement by nonvaccine serotypes [28]. Since the collection of middle ear fluid in this study was restricted to the first otitis media episode after the booster vaccination and the time interval between this vaccination and recurrence of otitis media was relatively short, these data match our present finding of a temporary increase in *S. aureus* carriage directly following the booster dose.

With respect to surveillance of invasive *S. aureus* disease, no substantial increase has been reported so far. A recent study on paediatric community-acquired bacteremia in children aged 0 to 15 years between 2001 and 2008 in Paris, France, where PCV7 was implemented in 2002 for infants under 2 years of age but with low uptake until 2005, reported no increase in *S. aureus* bacteremia in this period [29]. In the United Kingdom, where PCV7 was implemented in the national immunisation program in September 2006 with a catch-up campaign for children up to the age of 2 years, a similar study covering the period 1998 to 2007 showed a small increase in *S. aureus* bacteremia in infants aged 1 to 11 months in 2007 but a decrease in older children [30]. However, surveillance studies for severe staphylococcal infections in children for a longer period after widespread PCV-implementation are mandatory to observe the full impact on *S. aureus* epidemiology, particularly in the current era with multi resistant *S. aureus* strains and the introduction of broader coverage conjugate vaccines.

The prevalence of *S. aureus* colonisation in parents of both vaccinated and unvaccinated children was considerably higher than in children. The course of *S. aureus* colonisation, with high colonisation rates in infancy rapidly decreasing in childhood but again increasing towards adulthood, suggests that acquired immunity cannot fully explain the dynamics in *S. aureus* [8], [31]. Environmental factors, such as close contact between parents and young infants may explain the high *S. aureus* rates in young infancy [31]. Remarkably, *S. aureus* colonisation rates in parents decreased between the child's age of 12 and 24 months as well as rates for *S. pneumoniae*. The colonisation rates of parents at the child's age of 24 months, in particular that of unvaccinated children, resemble more the lower carriage rates previously found in young adult general population [8]. The route of transmission either from child to parent or vice versa cannot be distinguished from our data. A recent study by Regev-Yochay et al. demonstrated a strong association between child and parental *S. aureus* colonisation with the most likely route of transmission in their study from parent to child [32]. Our data rather suggest that some transmission from child to parent also occurs, in particular during the first years of life with high mutual exposure via close contact. The higher pneumococcal and *S. aureus* carriage rates in parents of vaccinated children compared to unvaccinated children reflect the increased dynamics of these bacteria after PCV7 vaccination in children in the second year of life [5].

Some limitations of our study should be recognized. First, this study was hypothesis driven. We did not correct for multiple testing and therefore results must be interpreted with caution. Furthermore, the rates for *S. aureus* in our study are low compared to several other studies [9], [22], [32]. This is likely related to the chosen sampling procedure for this replacement study, taking a nasopharyngeal swab from the posterior nasopharynx and not a separate nasal swab from the anterior nares, the ecological site for *S. aureus* [33]. The nasopharyngeal presence of *S. aureus* in our study was quite similar to that found by Cohen and colleagues who also only obtained a nasopharyngeal swab in children with otitis media [18]. Nevertheless, the applied method in our study was similar for all 3 groups and therefore the observed differences in *S. aureus* presence between vaccinees and controls are valid. Last, the full 4-dose schedule comprising 3 primary doses and a booster dose may have additional effect on shifts in nasopharyngeal colonisation compared with our study that evaluated the effects of reduced-dose schedules. Major strength of our study is the randomized, controlled and longitudinal study design in a PCV7-unvaccinated community in the absence of herd effects and with relatively low bacterial antibiotic resistance rates and therefore truly evaluating vaccine effects [34]. The effects of nationwide PCV-introduction and subsequent herd effects on *S. aureus* colonisation in young children need further monitoring.

In conclusion, we observed a temporary increase in *S. aureus* colonisation after PCV7 in young children still at a vulnerable age for bacterial infections. Widespread routine infant PCV7 vaccination may cause, apart from pneumococcal serotype shifts, other shifts in nasopharyngeal bacterial colonisation in the population. This may especially be relevant after implementation of new broader coverage vaccines, like the recently licensed 10-valent and 13-valent PCV's, and/or widespread implementation of the full 4-dose schedule which may induce more outspoken shifts in the nasopharyngeal flora compared with our study. The potential clinical consequences of the observed shifts in *S. aureus* colonisation are unknown and further evaluation and clinical surveillance is needed, particularly in the current era with multi resistant strains.

## Supporting Information

Checklist S1 **CONSORT checklist.**
(DOCX)

Protocol S1 **Trial protocol.**
(PDF)

Summary Protocol S1 **Summary trial protocol.**
(PDF)
