# Supplementary material for: Effect of Seven-Valent Pneumococcal Conjugate Vaccine on Staphylococcus aureus Colonisation in a Randomised Controlled Trial
Source: PLoS One. 2011 Jun 10;6(6):e20229. doi: 10.1371/journal.pone.0020229 (PMC3112202; doi:10.1371/journal.pone.0020229)
Supplement: Protocol S1 — Trial protocol. (PDF) [file pone.0020229.s002.pdf]

Invloed van 2 en 3 Prevenar® vaccinaties in het RVP op pneumokokken neusdragerschap, transmissie en 'herd-immunity': Een gerandomiseerd, gecontroleerd onderzoek.

Korte titel: Invloed van Prevenar op Nasopharyngeaal Dragerschap en Herd Immunity

Subsidiegever: Ministerie van Volksgezondheid, Welzijn en Sport
Opdrachtgever: Universitair Medisch Centrum Utrecht

Contactpersoon studie:
Prof Dr. EAM Sanders
Kinderarts-immunoloog
Wilhelmina Kinderziekenhuis/
Universitair Medisch Centrum Utrecht
Hp 03.063.0
Postbus 85090
3508 AB Utrecht
tel: 030 2504003
fax: 030 2505350
email: l.sanders@umcutrecht.nl

Protocol Autorisatie

Naam	Titel	Handtekening	Datum
	Ministerie van VWS		
Dr. L van Alphen	Productmanager meningokokken en pneumokokken vaccins NVI		
Prof Dr. BAM van der Zeijst	Wetenschappelijk Directeur NVI		
Prof Dr. EAM Sanders (auteur)	Principal Investigator		
Dr. RH Veenhoven	Projectleider		
Dr. E Hak	Klinisch Epidemioloog		

Onderzoeksgroep

- **Medewerkers onderzoek**

Lieke Sanders (Prof dr.)	Kinderarts immunoloog	Principle investigator, projectleider	Wilhelmina Kinderziekenhuis/UMC Utrecht	Uitgebreide ervaring met grootschalige trials op gebied van vaccinatie en luchtweginfecties bij kinderen
Reinier Veenhoven (Dr.)	Kinderarts	Projectleider	Spaarne Ziekenhuis Hoofddorp	Gepromoveerd op Nederlands onderzoek naar Prevenar® bij kinderen met oorontstekingen (OMAVAX)
Eelko Hak (Dr.)	Epidemioloog	Projectleider	Julius Centrum voor Huisartsgeneeskunde en Patientgebonden Onderzoek	Uitgebreide expertise met grootschalige studies naar effect van (influenza)vaccinaties
Arie van der Ende (Dr.)	Microbioloog	Serotypering IPD	Nationaal Referentie Laboratorium voor Bacteriële Meningitis	Expertise in epidemiologie en pathogenese van meningitis
Ed IJzerman (Dr.)	Microbioloog	Microbioloog	Hoofd Regionaal Laboratorium Microbiologie Haarlem	Microbioloog in OMAVAX onderzoek
Ger Rijkers (Dr. Ir)	Medisch immunoloog	Medisch Immunoloog	Hoofd laboratorium pediatrische immunologie WKZ/UMC Utrecht	Uitgebreide expertise in immunologisch pneumokokken onderzoek
Elske van Gils (Drs.)	Arts-onderzoeker		UMCUtrecht	Artsonderzoeker MINOES

Contactadressen

Subsidie voor Onderzoek wordt verstrekt door VWS
Contactpersoon in het Nederlands Vaccin Instituut (NVI) voor VWS
Dr. Loek van Alphen, Productmanager meningokokken- en pneumokokkenvaccins
Antonie van Leeuwenhoekslaan 11, 3721 MA Bilthoven
Nederland
Postbus 457, 3720 AL Bilthoven, Nederland
Telefoon 31 30 2742701
Telefax 31 30 2287874
Email loek.van.alphen@nvi-vaccin.nl, www.nvi-vaccin.nl

Prinicipal Investigator
Prof Dr. Lieke Sanders, kinderarts-immunoloog
Het Wilhelmina Kinderziekenhuis/ Universitair Medisch Centrum Utrecht
Lundlaan 6, 3508 AB, Utrecht, The Netherlands
Hp 03.063.0
Postbus 85090, 3508 AB Utrecht, The Netherlands
tel: 030 2504003
fax: 030 2505350
email: l.sanders@umcutrecht.nl

Projectleider
Dr. Reinier Veenhoven, kinderarts
Spaarne Ziekenhuis Hoofddorp
Spaarnepoort 1, 2134 TM Hoofddorp,
Nederland
Postbus 770, 2130 AT Hoofddorp, Nederland
Telefoon 31 23 8908900 of 8907414
Email rveenhoven@spaarneziekenhuis.nl

Onafhankelijk arts
Dr. Peter de Winter, kinderarts-neonatoloog
Spaarne Ziekenhuis Hoofddorp
Spaarnepoort 1, 2134 TM Hoofddorp,
Nederland
Postbus 770, 2130 AT Hoofddorp, Nederland
Telefoon 31 23 8908900
Email: pdewinter@Spaarneziekenhuis.nl

Klinisch epidemioloog
Dr. Eelko Hak
Universitair Medisch Centrum Utrecht
Julius Centrum voor Gezondheidswetenschappen en Huisartsgeneeskunde
HP 6.139
Postbus 85060, 3508 AB Utrecht, Nederland
Telefoon 31 30 253 8214
Telefax 31 30 253 9028
Email: e.hak@umcutrecht.nl, www.juliuscenter.nl

Arts-onderzoeker

Drs Elske van Gils
Arts-onderzoeker WKZ/UMCUtrecht
Onderzoekscentrum MINOES
Spaarne Ziekenhuis Hoofddorp
Spaarnepoort 1, 2134 TM Hoofddorp,
Nederland
Postbus 770,
2130 AT Hoofddorp, Nederland
Telefoon 31 23 8909070
Email: EvanGils@SpaarneZiekenhuis.nl

Synopsis

titel	Invloed van 2 versus 3 Prevenar® vaccinaties in het RVP op pneumokokken neusdragerschap, transmissie en 'herd-immunity': een gerandomiseerd, gecontroleerd onderzoek
Sponsor	UMC Utrecht
Trial fase	Fase 3b
Doelstelling	**Primaire doel:** vaststellen van de invloed van 2 en 3 pneumokokken conjugaatvaccinaties op de nasopharyngeale (NP) bacteriële kolonisatie van gevaccineerde zuigelingen<br>**Secundaire doelstellingen**<br>- Onderzoeken (NP dragerschap gezinscontacten) en voorspellen van effecten van 'herd-immunity'<br>- Onderzoeken welke vaccintype (VT) en non-vaccintype (NVT) pneumokokken in Nederland circuleren voorafgaand aan Prevenar® invoering<br>- Onderzoeken welke NVT pneumokokken opkomen na pneumokokken conjugaatvaccinaties<br>- Volgen van de relatie NP dragerschap en invasieve pneumokokkenziekten (IPD)<br>- Evaluatie van invloed op andere koloniserende species als *S. Aureus*, B. pertussis<br>Tevens<br>- Evalueren van eventuele reductie van community-acquired acute otitis media en lage luchtweginfecties als pneumonie na pneumokokken conjugaatvaccinaties<br>- Veiligheid vaccin
Studie rationale	Zowel een 3-doses vaccinatie schema op **2, 4 en 11** maanden als een 2-doses schema op **2 en 4** maanden geven bescherming tegen IPD met VT-pneumokokken. De invloed op NP dragerschap zou bij 2 vaccinaties beperkter kunnen zijn dan bij 3 vaccinaties. Dit heeft consequenties voor verschuivingen van bacteriële flora op nasopharyngeaal niveau zoals gezien na Prevenar® vaccinaties, voor effecten op de potentiële "herd-immunity" en ten aanzien van preventie van pneumokokken luchtweginfecties als otitis en pneumonie.
Studie opzet	gerandomiseerd, gecontroleerd onderzoek
Studie populatie	Alle nieuwgeboren van 2-4 weken oud die in aanmerking komen voor het RVP in de regio Haarlem e.o. Kinderen worden gevolgd tot de leeftijd van 24 maanden
Inclusie	Kinderen jonger dan 12 weken waarvan de ouders/wettelijk vertegenwoordigers

criteria	schriftelijk hebben toegestemd na mondelinge en schriftelijke informatie.
Exclusie criteria	Aanwezigheid van een ernstige ziekte die medische zorg vereist die kan interfereren met de resultaten van de studie, bekende of vermoede allergie/overgevoeligheid tegen een van de vaccinbestanddelen, bekende of vermoede afweerstoornis, tevoren toegediend zijn van plasmaproducten (inclusief immuunglobulinen), tevoren reeds gevaccineerd zijn (anders dan met hepatitis B vaccinatie), bloedingstoornissen.
Aantal benodigde deelnemers	1000 zuigelingen, die worden verdeeld in 3 gelijke groepen. Rekening houdend met terugtrekken van toestemming en drop-outs worden 330 zuigelingen per studie arm geïncludeerd. Voor neuskweken participeren tevens één van de ouders/verzorgers en (indien aanwezig) één van de broers/zussen van de zuigeling.
Vaccinatie schema	Groep I. Prevenar® op leeftijd 2 en 4 maanden<br>Groep II. Prevenar® op 2, 4 en 11 maanden<br>Groep III. Prevenar® op 24 maanden<br>Vaccinaties op 2, 4 en 11 maanden worden gelijktijdig met DaKTP-Hib (evt. Hep B) op het consultatiebureau gegeven.
Methode: **Huisbezoek** voor afname neuskweken en vragenlijsten	Afname van transnasale nasopharyngeale watten voor bacteriële kweek:<br>- Bij de zuigeling vóór de leeftijd van 2 maanden en vervolgens op 6, 12, 18 en 24 maanden leeftijd<br>- Als de zuigeling 12 en 24 maanden oud is bij één van de ouders en één van de broers/zussen. Afname van oropharyngeale watten voor bacteriële kweek bij dezelfde ouder<br>-Vragenlijsten op 2, 6, 12, 18 en 24 maanden met betrekking op risicofactoren voor pneumokokkendragerschap en doorgemaakte infecties.<br>- Bij doktersconsultatie opvragen van gegevens bij de huisarts en specialist
Bloed onderzoek	Bij 80 kinderen van de groep met 2 en 3 vaccinaties en 30 kinderen in de controle groep met Prevenar op 24 maanden worden op 12 en 24 maanden 3 ml bloed geprikt ter bepaling van anti-pneumokokken afweerstoffen (huisbezoek).
Onderzoeks vaccin	Geregistreerd 7-valent pneumokokken polysaccharide-eiwit conjugaatvaccin [Prevenar®]
Veiligheid	Melding adverse events, dagboekregistratie en temperatuursmeting na vaccinaties. Minimale risico's verbonden aan venapunctie en neuskweek.
Statistische analyse	Om een statistisch significant verschil te kunnen aantonen in de reductie van VT pneumokokken dragerschap bij 2 en 3 Prevenar® vaccinaties van 35% naar 25% van de pneumokokken isolaten uit de nasopharynx, moeten er bij een alfa van 5% en een onderscheidend vermogen van 80% (beta = 20%), 300 kinderen in iedere groep worden geïncludeerd. Rekening houdend met een uitval van 10%

	betekent dit een inclusie van 1000 zuigelingen in de studie.
Interim analyse	6 maanden nadat alle kinderen het volledige vaccinatieschema op 2, 4 en 11 maanden hebben doorlopen, zal een interim analyse worden verricht op de uitkomsten van de nasopharyngeale kweken en het eerste bloedonderzoek.

Invloed van 2 en 3 Prevenar® vaccinaties in het RVP op pneumokokken neusdragerschap, transmissie en 'herd-immunity': een gerandomiseerd, gecontroleerd onderzoek.

Doelstelling

Als Prevenar® wordt geïntroduceerd in het RVP voor zuigelingen op de leeftijd 2, 4 en 11 maanden, is Nederland één van de eerste Europese landen die landelijk gaat vaccineren tegen pneumokokken. In het jaar daaraan voorafgaand, is het mogelijk valide gegevens te verzamelen met betrekking tot nasopharyngeaal dragerschap en transmissie van pneumokokken bij zuigelingen als basis om kosteneffectiviteitmodellen als ook transmissiemodellen te onderbouwen voor de Nederlandse situatie. Transmissiemodellen geven inzicht in te behalen 'herd immunity' en beschikbare data zijn vooralsnog gebaseerd op gegevens in de VS waaronder incidentiegetallen van invasieve pneumokokkenziekten (IPD) na introductie van Prevenar® in de USA. [1-3] Momenteel zijn deze modellen niet empirisch onderbouwd met gegevens over nasopharyngeaal (NP) dragerschap en transmissie in de bevolking. De resultaten wijzen onverwacht op een groot 'herd immunity' effect op alle leeftijden, maar bij interpretatie van deze Noord-Amerikaanse trendstudies zonder controle groep is het onduidelijk welke invloed andere factoren kunnen hebben gehad als bijvoorbeeld toegenomen influenzavaccinatiegraad, verbeterd antibiotisch gebruik of natuurlijke pneumokokkenziekte fluctuaties in de tijd. [4,5] Omdat deze factoren in Nederland kunnen verschillen van die in de VS, kan ook de 'herd immunity' door Prevenar® in Nederland verschillen van die in de VS en is deze op langere termijn niet goed voorspelbaar.

Naast de noodzaak voor empirische onderbouwing van 'herd immunity', bestaat in Nederland een unieke mogelijkheid om de effecten van twee doses en drie doses Prevenar® op de 'herd immunity' te bestuderen en effecten op nasopharyngeaal dragerschap te vergelijken in een gecontroleerde studie. Aangenomen mag worden dat een 2-doses schema (op de leeftijd 2 en 4 maanden) beschermt tegen invasieve pneumokokkeninfecties als meningitis [6-8] en daarom kosteneffectief aantrekkelijk is voor het RVP in de toekomst. Echter, een 2-doses schema geeft potentieel minder afname van vaccintype (VT) pneumokokken dragerschap, hetgeen betekent dat minder 'herd-immunity' mag worden verwacht en minder bescherming tegen pneumonie en oorontstekingen. Een hypothetisch voordeel van minder invloed van een 2-doses schema op de nasopharyngeale flora is een verlaagde kans op potentieel negatieve effecten van pneumokokken conjugaatvaccinaties zoals het opkomen van niet-vaccintype (NVT) pneumokokken [9-12] alsmede toegenomen dragerschap van andere bacteriële species als *Staphylococcus aureus* [11, 13, 14] en *Neisseria meningitidis*. [15]
Om de bestaande gezondheidseconomische en transmissiemodellen te kunnen optimaliseren voor de Nederlandse situatie en de effecten van 2 versus 3 vaccinaties te kunnen schatten met betrekking tot het optreden van 'herd immunity', is onderzoek naar de effecten op de nasopharyngeale flora van gevaccineerde zuigelingen en directe contacten in het gezin van het grootste belang voorafgaand aan en volgend op landelijke introductie van pneumokokken vaccinatie. Zoals internationaal wordt

aanbevolen vormen de gegevens een essentiële basis voor het monitoren van lange-termijn effecten. [16] Tenslotte zullen de resultaten van belang zijn bij andere strategieën zoals bij vaccinatie tegen meningokok B, een van de vaccins welke in de nabije toekomst naar verwachting eveneens zal worden geïntroduceerd in het RVP.

Vraagstelling van de studie

- Wat is de invloed van 2 en 3 pneumokokken conjugaatvaccinaties op de NP bacteriële kolonisatie van gevaccineerde zuigelingen en gezinscontacten?
- Welk inzicht geeft dit in het voorspellen van het effecten van 'herd-immunity'?

- Opportunities in relatie tot pneumokokkenconjugaatvaccinaties

- Welke VT en NVT serotypen pneumokokken circuleren in Nederland voorafgaand aan Prevenar® invoering?
- Welke NVT serotypen worden gezien na pneumokokken conjugaatvaccinaties?
- Hoe is de relatie NP dragerschap en IPD?
- Is er na pneumokokken conjugaatvaccinaties bij de gevaccineerde zuigeling een reductie van community-acquired acute otitis media en lage luchtweginfecties pneumonie waarneembaar?
- Wat zijn de antistoftiters na 2 versus 3 vaccinaties op 12 maanden (1 maand na booster op 11 maanden) en op 24 maanden?

- Opportunities in relatie tot toekomstige vaccinaties

- Hoe is de relatie met andere koloniserende en in potentie pathogene species als *Staphylococcus aureus* ?
- In welke frequentie circuleert *Bordetella pertussis* bij kinderen tussen 2 en 24 maanden in relatie tot kinkhoestvaccinaties in het RVP (met name ten tijde van epidemieën) ?

_ Registratie

- De gegevens voortkomend uit deze studie kunnen worden gebruikt bij het aanvragen van registratie van 2 doses Prevenar voor de leeftijd van 6 maanden van een zuigeling, met vervallen van de derde booster dosis op 11 maanden.

Studie opzet

Inclusie van 1000 pasgeborenen, in het jaar voorafgaand aan landelijke implementatie van Prevenar®

- Werving

Via de entadministratie krijgen alle ouders van alle nieuwgeborenen in de leeftijd van 2-4 weken (n=16.500 per jaar) van de consultatiebureau regio's in de omgeving van Haarlem (regio's Zuid-Kennemerland, IJmond, Amstelring, Groot Rijnland, Valent RDB en Florence) een uitnodigingsbrief met schriftelijk informatie over de studie toegestuurd (bijlage 1). Wanneer ouders belangstelling hebben, wordt hen gevraagd de bijgevoegde portvrije antwoordkaart terug te sturen met daarop vermeld hun telefoonnummer. Na telefonisch contact met een van de onderzoeksmedewerkers krijgen

zij dan per post uitgebreidere informatie en informed consent formulieren toegestuurd (bijlage 2). Na 7 dagen worden zij opnieuw telefonisch benaderd of zij meedoen en indien akkoord wordt een eerste afspraak gemaakt voor een huisbezoek. Bij dit eerste huisbezoek wordt een vragenlijst afgenomen met betrekking tot risicofactoren voor verwerven van pneumokokken dragerschap (o.a. gezinssamenstelling, roken, zwangerschapsduur, geboortegewicht, borstvoeding, crèche bezoek). Indien geen contra-indicaties voor de studie bestaan en zodra het schriftelijk informed consent door beide ouders en een bevoegde onderzoeksmedewerker is getekend, wordt de zuigeling vervolgens gerandomiseerd in een van de 3 vaccinatie groepen. Tevens wordt dan de eerste transnasale nasopharyngeale kweek afgenomen bij de zuigeling. De ouders wordt een brief overhandigd met gegevens over het onderzoek en de indeling in de groep van de betreffende zuigeling. Deze brief nemen zij mee voor bezoek op het consultatiebureau op de leeftijd 2 maanden. De CB arts geeft dan de eerste vaccinaties (DaKTP-Hib (evt. hepatitis B)) ± Prevenar® . Tevens krijgen ouders een informatiebrief mee voor de huisarts.

Behalve benadering via de entadministratie zal ook met folders en mondelinge informatie worden geworven via verloskundigen, consultatiebureaus en huisartsen. Informatieavonden zullen worden georganiseerd voor verloskundigen, CB-medewerkers en huisartsen. Tevens zal met persberichten verdere bekendheid aan het onderzoek worden gegeven.

- **Vaccinatiegroepen en controle**

De vaccinaties vinden plaats op het CB gelijktijdig met de overige RVP vaccinaties door de wijkverpleegkundigen JGZ danwel de CB-arts.

Groep I. Prevenar® op leeftijd 2 en 4 maanden, gelijktijdig met DaKTP-Hib (evt. Hep B)
Groep II. Prevenar® op 2, 4 en 11 maanden, gelijktijdig met DaKTP-Hib (evt. Hep B)
Groep III. Prevenar® op 24 maanden

- **Follow-up**

Gedurende een follow-up periode van twee jaar vinden huisbezoeken plaats door een getrainde onderzoeksmedewerk(st)er op de zuigelingenleeftijd van 6, 12, 18 en 24 maanden. Tijdens dit huisbezoek worden afgenomen:

- Een nasopharynxkweek bij de zuigeling op leeftijd 6, 12, 18 en 24 maanden;
- Een nasopharynxkweek van één van de ouders/verzorgers en één van de broers/zussen (indien aanwezig) op de zuigelingenleeftijd 12 en 24 maanden;
- Een oropharynxkweek bij dezelfde ouder/verzorger bij het huisbezoek op de leeftijd 12 en 24 maanden van de zuigeling;
- Een vragenlijst met betrekking tot omgevingsfactoren met invloed op dragerschap pneumokok van allen bij wie een kweek wordt afgenomen

- **Venapunctie**

Aan ouders/verzorgers wordt toestemming gevraagd voor een vrijwillige en aanvullende venapunctie bij de zuigeling op de leeftijd 12 en 24 maanden voor onderzoek naar afweerstoffen met betrekking tot

de Prevenar® vaccinaties (3 milliliter per afname). De venapunctie vindt plaats door een ervaren artsonderzoeker. In de groepen met 2 danwel 3 vaccinaties vindt venapunctie plaats bij 80 kinderen per groep, in de controle groep met eenmalige vaccinatie op 24 maanden bij 30 kinderen. Dit betekent in totaal bij 190 kinderen.

- **Pneumokokken infecties**

Invasieve pneumokokken meningitis oorzaken en serotypen worden vastgelegd via het reeds bestaande landelijke registratie systeem via (Nationaal Referentielaboratorium voor Bacteriële Meningitis, Amsterdam). Meningitis-isolaten worden bewaard voor onderzoek.
Gegevens met betrekking tot artsbezoeken, oorontstekingen, luchtweginfecties, antibioticagebruik en evt. ziekenhuisopnamen worden na toestemming van de ouders via de huisarts of specialist opgevraagd.

- **Te verwachten effecten**

Na de leeftijd van 6 maanden loopt het pneumokokkendragerschap bij ongevaccineerde zuigelingen op tot tenminste 50%. Hiervan is de helft van de serotypen geïncludeerd in Prevenar® volgens recent Nederlands onderzoek, indien geen rekening wordt gehouden met kruisreactieve serotypen als serotype 6A. [13] Na implementatie van de 3 vaccinaties op 2, 4 en 11 maanden verwacht men op de leeftijd 12 maanden dat het dragerschap van VT- pneumokokken met tenminste 50% is gedaald ten opzichte van de ongevaccineerde groep, met gelijktijdige opkomst van NVT serotypen [9,11,12] en waarschijnlijke verschuivingen van diverse andere nasopharyngeale bacteriële species (stafylokokken, streptokokken,..). [13-15]
Indien slechts twee vaccinaties worden gegeven op de leeftijd 2 en 4 maanden is er wel bescherming tegen invasieve infecties (IPD) met VT-pneumokokken maar potentieel verminderde reductie van nasopharyngeaal dragerschap ten opzichte van 3 vaccinaties (geschatte reductie van 33% ten opzichte van de controle groep). Er wordt vanuit gegaan dat de effecten van 3 vaccinaties identiek zijn aan eerdere studies met 4 vaccinaties en een reductie van 50% wordt gezien op de leeftijd van 12 maanden.

- **Groepsgrootte berekening.**

Om een statistisch significant verschil te kunnen aantonen in de reductie van VT pneumokokken dragerschap bij 2 en 3 Prevenar® vaccinaties van 35% naar 25% van de pneumokokkenisolaten uit de nasopharynx, moeten er bij een alfa van 5% en onderscheidend vermogen van 80% (beta = 20%), 300 kinderen in iedere groep worden geïncludeerd. Rekening houdend met een uitval van 10% betekent dit een inclusie van 1000 zuigelingen in de studie.

- **Herd-immunity**

De consequentie van verminderde reductie van VT-pneumokokken dragerschap is verminderde reductie van transmissie en minder ‘herd-immunity’. Berekening van de kosteneffectiviteit met effecten van ‘herd-immunity’ in het model van Bos [17] is gebaseerd op aannames van IPD gegevens in de VS

na introductie van Prevenar®. Door gegevens over dragerschap en transmissie in ons onderzoek met 2 en 3 Prevenar vaccinaties te vergelijken met andere dragerschapstudies (Finland, Israël, USA, Zuid-Afrika) en dit te interpreteren in het licht van te verwachten 'herd-immunity' in de Nederlandse situatie, krijgen we nauwkeuriger indicatie wat te verwachten bij 2 en 3 doses Prevenar®. De gegevens kunnen zo gezondheidseconomische en transmissiemodellen optimaliseren voor de Nederlandse situatie. Tenslotte kan deze kennis worden toegepast bij andere strategieën zoals vaccinatie tegen meningokok B, welke in de nabije toekomst naar verwachting eveneens zal worden geïntroduceerd in het RVP.

- **Lange termijn monitoren**

Gezien het opkomen van NVT pneumokokken dragerschap na conjugaatvaccinaties en effecten op andere bacteriële species, vormen de gegevens uit dit onderzoek een goede basis voor lange termijn monitoren van de effecten van pneumokokken conjugaatvaccinaties in het RVP.

- **Haalbaarheid**

In de regio's Zuid-Kennemerland, IJmond, Amstelring, Groot Rijnland, Valent RDB en Florence vinden ± 16.500 bevallingen plaats per jaar. Bij een inclusieperiode van 7,5 maand worden ± 10.300 ouders aangeschreven. Indien 1: 10 ouders toestemt in participatie aan het onderzoek wordt de inclusie gehaald. Indien van de deelnemende kinderen 1 : 4 ouders toestemming geeft voor venapuncties bij kinderen uit groep I en II en 1:10 ouders van baby's in de controle groep, worden bij 190 kinderen de immunologische bepalingen uitgevoerd.

Achtergrond en literatuur

Pneumokokken behoren tot de normale microbiële flora van de nasopharynx (NP). In het eerste levensjaar stijgt het NP dragerschap tot 50% of meer en blijft gedurende de eerste 3 levensjaren hoog, om daarna geleidelijk af te nemen tot een stabiele kolonisatie rond 10-20% na het 10e levensjaar. [13,18] NP dragerschap van zuigelingen en jonge peuters vormt een belangrijke bron voor verspreiding van de pneumokok in het gezin en de bevolking.[1-3,17,22] Meestal is pneumokokken dragerschap asymptomatisch maar kan leiden tot ernstige infecties van de luchtwegen als longontsteking, oorontsteking en invasieve infecties als hersenvliesontsteking (meningitis).

Pneumokokken conjugaatvaccinaties leiden tot een reductie van NP dragerschap van pneumokokken serotypen welke zijn opgenomen in het vaccin (VT pneumokokken). [9-12,19, 20] Dit verminderd dragerschap wordt gezien bij zowel het gevaccineerde kind maar ook bij personen in de directe omgeving (gezin) en in de bevolking.[20,22] Dit leidt tot afname van pneumokokken infecties bij ongevaccineerde personen, een fenomeen beschreven als 'herd-immunity'. [1-3] Het betreft echter postmarketing trendstudies in de Verenigde Staten zonder controlegroep waardoor het onmogelijk is na te gaan of andere factoren als natuurlijke fluctuaties van IPD in de tijd hebben bijgedragen.[4,5] Eveneens is van belang dat gegevens over de gesuggereerde 'herd immunity' in de VS niet zijn onderbouwd met gegevens over NP dragerschap in de bevolking voor en na introductie van richtlijnen in 2000 om zuigelingen te vaccineren tegen pneumokokken.

Recent is aangetoond dat 3 doses Prevenar vrijwel even effectief lijken ter preventie van IPD op zuigelingen leeftijd als 4 doses (95% versus 97%).[2,7] Een definitieve registratiewijziging voor een 3-doses schema in Europa wordt daarom verwacht (op dit moment bij de EMEA in behandeling). Zelfs twee doses in de eerste zuigelingen maanden lijken voldoende bescherming te geven voor IPD.[6,7] Hoewel aantrekkelijk vanuit gezondheidseconomisch perspectief, is de vraag is of 2 doses in de eerste zuigelingen maanden ook leidt tot afname van NP dragerschap van VT-pneumokokken. Eerder Nederlands onderzoek liet zien dat bij oudere kinderen boven 1 jaar minimaal 2 conjugaatvaccinaties essentieel waren voor reductie van VT pneumokokken dragerschap. [12] Jonge zuigelingen van 2 en 4 maanden hebben gemiddeld gesproken nog geen eerdere pneumokokken contacten doorgemaakt en daarom is een derde booster vaccinatie wellicht noodzakelijk voor voldoende mucosale antistofvorming en reductie van dragerschap op nasopharynxniveau. Het niet beïnvloeden van VT-pneumokokken dragerschap zou betekenen dat herd-immunity niet optreedt en het effect van vaccinaties op luchtweginfecties en oorontstekingen afneemt. Een theoretisch voordeel van het niet beïnvloeden van NP flora is dat verschuiving van VT pneumokokken naar niet-vaccintype (NVT) pneumokokken niet zal optreden. Immers, duidelijk is in diverse studies dat afname van VT pneumokokken dragerschap gepaard gaat met een toename van NP dragerschap van NVT pneumokokken, waardoor het totale pneumokokken dragerschap niet verandert. [9,11,12] De vraag wat de impact van deze toename van NVT pneumokokken op de morbiditeit zal zijn is vooralsnog niet beantwoord en moet blijken in lange termijn vervolgstudies na introductie van het conjugaatvaccin zowel op NP niveau als naar verwekkers van IPD en luchtweginfecties. In een Finse studie naar

oorontstekingen bij zuigelingen na 4 conjugaatvaccinaties namen oorontstekingen door NVT pneumokokken toe met 33% bij een 57% afname van oorontstekingen door VT pneumokokken.[21] In de VS is na registratie van Prevenar® de toename van NVT-IPD weliswaar significant geworden, [3] maar in aantallen nog zo laag dat het grote profijt van de afname van VT- IPD niet wordt overschaduwd. Het monitoren van veranderd pneumokokkendragerschap en het optreden van IPD is echter vereist na introductie van het pneumokokken conjugaatvaccin. Het is bovendien noodzakelijk om in deze studies naaste contacten van de gevaccineerde zuigelingen te betrekken om de indirecte effecten van vaccinatie te kunnen evalueren.

Behalve de opkomst van NVT-pneumokokken kan het pneumokokken conjugaatvaccin ook de NP kolonisatie van andere species beïnvloeden, hoewel dit nog nauwelijks is bestudeerd. In een eigen Nederlandse studie bij kinderen met recidiverende oorontstekingen is echter aangetoond dat isolatie van *Staphylococcus aureus* uit middenooreffusie bij oorontstekingen significant toenam na pneumokokken conjugaatvaccinatie. [11] Interactie van *S. aureus* en VT- pneumokokken is ook geobserveerd in de Nederlandse AHOY studie bij 3100 kinderen van 1-18 jaar waarbij een afname van NP pneumokokken dragerschap was gekoppeld aan een toename van *S. aureus* kolonisatie. [13] Deze bevinding is inmiddels elders bevestigd. [14] Interspecies competitie tussen bijvoorbeeld alfa hemolytische streptokokken en pneumokokken, *Haemophilus influenzae* en *Moraxella catarrhalis*, alle gewone kolonisanten van de nasopharynx, is beschreven evenals potentiële invloed op *Neisseria meningitidis*. [14] De effecten op NP dragerschap lijkt dus van het grootste belang voor onderzoek en monitoren bij introductie van Prevenar® in het RVP.

- **Literatuur**

1. Whitney CG, Farley MM, Hadler J, Harrison LH, Bennett NM, Lynfield R, Reingold A, Cieslak PR, Pilishvili T, Jackson D, Facklam RR, Jorgensen JH, Schuchat A. Active Bacterial Core Surveillance of the Emerging Infections Program Network. Decline in invasive pneumococcal disease after the introduction of protein-polysaccharide conjugate vaccine. N Engl J Med. 2003;348:1737-46

2.Black S, Shinefield H, Baxter R, Austrian R, Bracken L, Hansen J, Lewis E, Fireman B. Postlicensure surveillance for pneumococcal invasive disease after use of heptavalent pneumococcal conjugate vaccine in Northern California Kaiser Permanente. Pediatr Infect Dis J 2004;23:485-9

3. CDC.Confidential preliminary CDC-update, presented at 4th International symposium on Pneumococci and Pneumococcal disease. May 9-13, 2004. Helsinki, Finland

4. Konradsen HB, Nokleby H. The conjugate vaccine and invasive pneumococcal disease. New Eng J Med 2003 August 14; 349(7): 714- 715

5. Rubin I, Shafinoori S, Ginocchio CC, Greenberg AJ, Yeoman E, Cheddie M. Impact of the severity of winter Influenza-like Illnesses and heptavalent pneumococcal conjugate vaccine in invasive pneumococcal infections in children and adults at a health care system in New York. 4th International symposium on Pneumococci and Pneumococcal disease. May 9-13, 2004. Helsinki, Finland. Abstract EPI-72

6. Goldblatt D, Ashton L, Southern J, Burbidge P, Burrage M, Morris R, Borrow R, Cartwright K, Miller E. Immunogenicity and boosting following a reduced number of doses of a pneumococcal conjugate vaccine in infants and toddlers. 4th International symposium on Pneumococci and Pneumococcal disease. May 9-13, 2004. Helsinki, Finland. Abstract New-06

7. Whitney C. Effect of pneumococcal conjugate vaccine on invasive disease in the U.S. 4th International symposium on Pneumococci and Pneumococcal disease. May 9-13, 2004. Helsinki, Finland. Oral presentation, Abstract PSV1-02

8. Lucero MG, Puumalainen T, Ugpo JM, Williams G, Kayhty H, 7.Nohynek H. Similar antibody concentrations in Filipino infants at age 9 months, after 1 or 3 doses of an adjuvanted, 11-valent pneumococcal diphtheria/tetanus-conjugated vaccine: a randomized controlled trial.J Infect Dis 2004;189:2077-84

9. Mbelle N, Huebner RE, Wasas AD, Kimura A, Chang I, Klugman KP. Immunogenicity and impact on nasopharyngeal carriage of a nonavalent pneumococcal conjugate vaccine. J Infect Dis 1999; 180:1171-6

10. Dagan R, Givon-Lavi N, Janco J, et al. Reduction of nasopharyngeal carriage of *Streptococcus pneumoniae* after administration of a 9-valent pneumococcal conjugate vaccine to toddlers attending day care centers. J Infect Dis 2002; 927-36

11. Veenhoven RH, Bogaert D, Uiterwaal CSPM, Brouwer CNM, Kiezebrink HH, Hermans PWM, Zegers BJM, Kuis W, Ger Rijkers GT, Schilder AGM, Sanders EAM. Effect of conjugate pneumococcal vaccine followed by polysaccharide vaccine on recurrent acute otitis media. The Lancet, 2003;361:2198-05

12. Veenhoven RH, Bogaert D, Schilder AGM, Rijkers GT, Uiterwaal CSPM, Kiezebrink HH, van Kempen MJP, Dhooge IJ, Bruin J, IJzerman EPF, de Groot R, Kuis W, Hermans PWM, Sanders EAM. Nasopharyngeal pneumococcal carriage after combined pneumococcal conjugate and polysaccharide vaccination in children with a history of recurrent acute otitis media. Clin Infect Dis 2004;39(7):911-9

13.Bogaert D, van Belkum A, Sluijter M, Luijendijk A, de Groot R, Rumke HC,
Verbrugh HA, Hermans PW. Colonisation by Streptococcus pneumoniae and Staphylococcus aureus in healthy children. Lancet 2004;363:1871-2.

14. Regev-Yochay G, Dagan R, Raz M, Carmeli Y, Shainberg B, Derazne E, Rahav G,
Rubinstein E. Association between carriage of Streptococcus pneumoniae and Staphylococcus
aureus in Children. JAMA. 2004;292:716-20

15. Pericone CD, Overweg K, Hermans PW, Weiser JN. Inhibitoryand bactericidal effects of hydrogen peroxide production by Streptococcus pneumoniae on other inhabitants of the upper respiratory tract. Infect Immun 2000;68(7):3990-72

16. O'Brien KL, Nohynek H, and the WHO pneumococcal vaccine trial carriage working group. Report from a WHO working group; standard method for detecting upper respiratory carriage of streptococcus pneumoniae. Pediatr. Infect Dis J 2003;22:133-139

17. Bos J.M. Kosten-effectiviteit van Prevnar: de effecten van update van gegevens, inclusie van herd immunity en vaccin-toediening op 2, 3, en 11 maanden. Confidential Report RIVM, juni 2004.

18. Syrjanen RK, Kilpi TM, Kaijalainen TH, Herva EE, Takala AK. Nasopharyngeal carriage of Streptococcus pneumoniae in Finnish children younger than 2 years old. J Infect Dis 2001; 184:451-9
19.Obaro SK, Adegbola RA, Banya WAS, Greenwood BM. Carriage of pneumococci after pneumococcal vaccination. Lancet 1996; 348:271-2
20.Dagan R, Muallem M, Melamed R, Leroy O, Yagupsky P. Reduction of pneumococcal nasopharyngaeal carriage in early infancy after immunization with tetravalent pneumococcal vaccines conjugated to either tetanus toxoid or diphteria toxoid. Pediatr Infect Dis J 1997; 16:1660-4
21. Eskola J, Kilpi T, Palmu A, et al. Efficacy of a pneumococcal conjugate vaccine against acute otitis media. N Engl J Med 2001; 344:403-9.
22. Givon-Lavi N, Fraser D, Dagan R. Vaccination of day-care center attendees reduces carriage of Streptococcus pneumoniae among their younger siblings. Pediatr Infect Dis J 2003;22:524-32

Studie opzet en procedures

- **Doel van de studie**

In de huidige studie worden nasopharyngeaal dragerschap van VT en NVT pneumokokken vergeleken van zuigelingen tot de leeftijd 24 maanden die twee keer (2 & 4 maanden) dan wel drie keer (2, 4 & 11 maanden) gevaccineerd zijn met Prevenar® en een ongevaccineerde controlegroep. De uitkomsten dienen als basis om verwachte 'herd-immunity' effecten en dus kosteneffectiviteit te onderbouwen voor de Nederlandse situatie. De dragerschapgegevens van ongevaccineerde zuigelingen dienen als basis om lange termijn effecten na implementatie van Prevenar® in het RVP te monitoren. Interacties met andere nasopharyngeaal koloniserende bacteriën worden met en zonder Prevenar® vaccinaties bestudeerd. Daarnaast worden antistoftiters van het 2-doses en 3-doses schema gecontroleerd en vergeleken op de leeftijd 12 maanden en 24 maanden.

- **Studie opzet**

Gecontroleerde, gerandomiseerde studie bij zuigelingen. Vergelijken van nasopharyngeaal pneumokokken dragerschap bij ongevaccineerde zuigelingen met zuigelingen die twee keer (leeftijd 2 en 4 maanden) en drie keer Prevenar® (leeftijd 2, 4 en 11 maanden) gelijktijdig met DaKTP-Hib ± Hep B krijgen. De studie wordt uitgevoerd in de regio Haarlem e.o.

**Groepsindeling**
Groep I. Prevenar® op de leeftijd 2 en 4 maanden,
Groep II. Prevenar® op 2, 4 en 11 maanden
Groep III. Prevenar® op 24 maanden

De Prevenar® vaccinaties op 2, 4 en 11 maanden vinden plaats door de CB-artsen of wijkverpleegkundigen JGZ gelijktijdig met de overige RVP vaccinaties (DaKTP-Hib en evt. Hepatitis B). De vaccinatie op 24 maanden wordt gegeven tijdens het laatste huisbezoek op 2 jaar door een bevoegd onderzoeksmedewerker.

Prevenar (0,5 ml) wordt intramusculair toegediend in het andere bovenbeentje dan waar DaKTP-Hib is gegeven. Indien tevens een eventuele Hep. B vaccinatie is gegeven in een bovenbeentje, dan wordt Prevenar i.m. toegediend in een bovenarmpje.

Studie eindpunten

- Primaire eindpunt

- Pneumokokken VT dragerschap bij zuigelingen en hun gezinsleden na 2 en 3 Prevenar® vaccinaties in vergelijking met een (tot 2 jaar) ongevaccineerde controlegroep.

- Secundaire eindpunten met betrekking tot pneumokokken vaccinaties

- Welke VT en NVT pneumokokken serotypen circuleren in Nederland voorafgaand aan de landelijke implementatie van Prevenar®
- Welke NVT pneumokokken komen op na vaccinaties
- Wat zijn de antistoftiters op 12 en 24 maanden bij Prevenar® vaccinatie op 2 & 4 maanden versus 2, 4 & 11 maanden?
- Hoe is de relatie tussen NP dragerschap en IPD in Nederland
- Is er een reductie in bezoeken aan een arts bij Prevenar®-gevaccineerde kinderen met betrekking tot luchtweginfecties (otitis media, pneumonie)
- Welk effect heeft de invloed op NP dragerschap op de te verwachten landelijke herd-immunity na implementatie van Prevenar® voor zuigelingen

- Overige eindpunten

- Hoe is de relatie met andere koloniserende species als *Staphylococcus aureus, Haemophilus influenzae, Moraxella catharalis, ß-haemolytische Streptokokken*
- In welke frequentie circuleert *Bordetella pertussis* bij kinderen tussen 2 en 24 maanden in relatie tot kinkhoestvaccinaties in het RVP (met name ten tijde van epidemieën)

Studie procedures

Alle zuigelingen van 2 tot 4 weken oud worden via de entadministratie benaderd voor deelname aan het onderzoek. Indien ouders/verzorgers belangstelling hebben (antwoordkaart teruggestuurd) voor het onderzoek wordt telefonisch nadere informatie verstrekt en uitgebreidere schriftelijke informatie en het informed consent formulieren toegestuurd. Na 7 dagen wordt opnieuw gebeld met ouders/verzorgers en bij positief antwoord een eerste huisbezoek afgesproken voordat de zuigeling 2 maanden oud is.

Bij dit eerste bezoek door een bevoegde onderzoeksmedewerk(st)er wordt een vragenlijst doorlopen. Indien geen contra-indicaties voor het onderzoek bestaan en door beide ouders/wettelijk vertegenwoordigers en onderzoeker het informed consent getekend is, wordt de baby gerandomiseerd in een van de drie vaccinatiegroepen. De eerste transnasale nasoharyngeale kweek wordt afgenomen. De ouders krijgen een formulier mee voor het consultatiebureau bij het bezoek op 2 maanden leeftijd voor de eerste DaKTP-Hib vaccinatie. Afhankelijk van de randomisatiegroep krijgt de zuigeling op 2, 4 en 11 maanden naast de DaKTP-Hib en eventuele Hepatitis B vaccinatie, een Prevenar® vaccinatie. Na afloop van de vaccinaties op 2, 4 en 11 maanden houden alle ouders van de deelnemende kinderen gedurende 7 dagen een dagboekje bij met bijwerkingen en meten 7 dagen eenmaal daags rectaal de temperatuur

Vervolgens vinden op zuigelingen leeftijd 6, 12, 18 en 24 maanden opnieuw huisbezoeken plaats. Tijdens deze bezoeken wordt een vragenlijst afgenomen met betrekking tot omgevingsfactoren die het dragerschap van pneumokokken kunnen beïnvloeden (borstvoeding, roken in huis, broers/zussen etc)

en met betrekking op eventuele consulten van de huisarts of een specialist in verband met infecties of opnamen in het ziekenhuis. Op 6, 12, 18 en 24 maanden worden transnasale nasopharyngeale kweken afgenomen bij de zuigeling. Op 12 en 24 maanden worden korte vragenlijsten afgenomen bij één van de ouders en een eventuele broertje/zusje bij wie dan ook transnasale nasopharyngeale kweken worden afgenomen. Bij de ouder wordt ook een oropharyngeale kweek afgenomen op de zuigelingenleeftijd van 12 en 24 maanden. Bij 1 : 4 van alle deelnemende kinderen met 2 of 3 doses Prevenar® worden na aparte toestemming op 12 en 24 maanden tevens 3 milliliter bloed (totaal 6 ml) afgenomen via een venapunctie, tijdens het huisbezoek door de artsonderzoeker of eventueel op de polikliniek van het Spaarne Ziekenhuis. Hetzelfde bij 1 : 10 kinderen van de controlegroep die eenmalig worden gevaccineerd op 24 maanden.

De huisarts krijgt bericht via een brief voor de ouders over deelname aan het onderzoek en de indeling in de groep met betrekking tot de vaccinaties.
Indien een consult van de huisarts dan wel specialist heeft plaatsgevonden gedurende de twee jaar follow-up worden de gegevens opgevraagd en geanalyseerd in relatie tot al dan niet gekregen Prevenar® vaccinaties.

Een gedetailleerde studieprocedure wordt beschreven.

- Pre-screening

- Alle ouders van baby's tussen 2 en 4 weken oud, geboren in het jaar voorafgaand aan Prevenar® invoering in het RVP in de omgeving van de consultatiebureaus Haarlem (Zuid-Kennemerland, IJmond, Amstelring, Groot Rijnland, Valent RDB en Florence) krijgen via de entadministratie een informatiefolder toegestuurd die kort het doel en de procedures van het onderzoek beschrijft. (zie bijlage 1) Ouders worden gevraagd een bijgevoegde portvrije antwoordkaart naar het onderzoeksteam terug te sturen met aangekruist of zij belangstelling hebben en hun telefoonnummer
- Ouders worden door onderzoeksmedewerkers gebeld om vragen over het onderzoek te beantwoorden en procedures uit te leggen
- Indien gewenst worden uitgebreidere schriftelijke informatie en een informed consent formulieren toegestuurd (zie bijlage 2)
- Na 7 dagen worden ouders opnieuw gebeld en bij positief antwoord wordt een eerste huisbezoek afgesproken vóór de baby de leeftijd 2 maanden heeft bereikt

- Eerste huisbezoek

- De toegestuurde informatie wordt doorgenomen en vragen worden beantwoord
- Een vragenlijst wordt afgenomen
- In- en exclusiecriteria worden gechecked
- Beide ouders/wettelijk vertegenwoordigers en de onderzoeksmedewerker tekenen informed consent
- Ouders krijgen kopie informed consent

- Na tekenen informed consent wordt de baby gerandomiseerd in een van de drie groepen.
- De baby krijgt een uniek onderzoek nummer (unique trial number, UTN) wat op elk onderzoeksformulier moet worden vermeld
- Transnasale nasopharyngeale kweek wordt afgenomen bij zuigeling
- Ouders krijgen een brief en formulieren mee met het UTN nummer voor het consultatiebureau mee. Hierin staat het vaccinatieschema voor de zuigeling
- Inentingskaarten Prevenar® worden uitgedeeld aan ouders
- Ouders krijgen een brief met informatie voor de huisarts mee
- Ouders krijgen dagboekjes voor 7 dagen na de vaccinaties op 2 en 4 maanden
- Ouders wordt de rectale temperatuursmeting uitgelegd
- Ouders worden geïnstrueerd contact op te nemen in geval van ziekenhuisopname/ SAE
- Huisbezoek op de leeftijd van 6 maanden wordt afgesproken
- Ouders wordt nogmaals gewezen op het onderzoekstelefoonnummer in geval van vragen of problemen
- Kweek wordt dezelfde dag ingezet op laboratorium microbiologie

- Consultatiebureau bezoek 2 en 4 maanden

- De baby krijgt - behalve de DaKTP-Hib ± Hep. B vaccinaties – afhankelijk van het onderzoeksvaccinatieschema Prevenar®, 0.5 ml i.m. in een andere ledemaat
- Na vaccinatie blijft de baby 15 minuten op het CB. Ouders observeren dan eventuele bijzondere reacties van de baby en waarschuwen een CB- medewerker indien directe lokale of systemische reacties op de vaccinaties worden gezien.
- De CB arts vult het onderzoeksformulier/ inentingskaart Prevenar® in met vermelden van het batchnummer en handtekening. Aangegeven wordt welke vaccinaties zijn gegeven en in welke ledemaat.
- De CB-arts/JGZ-verpleegkundige vult de gegevens in op de algemene inentingskaart voor de ouders, zet aantekening MINOES op DaKTP-kaart voor registratie entadministratie (indien gewenst), en in het eigen systeem
- De ouders houden gedurende 7 dagen volgend op de vaccinaties op 2 en 4 maanden een bijwerkingen dagboek bij, met eenmaal daags rectale temperatuur vermelding gedurende 7 dagen (dag 1 is de dag van vaccinatie)
- Ouders sturen dagboekje en inentingskaart op naar het onderzoekscentrum. In geval van onduidelijkheden/ problemen wordt contact opgenomen met de ouders

- Huisbezoek 2, leeftijd 6 maanden zuigeling

- Dagboekje voor 11 maanden wordt uitgereikt
- Vragenlijst wordt ingevuld
- Transnasale nasopharyngeale kweek wordt afgenomen bij zuigeling
- Afspraak voor huisbezoek 12 maanden wordt gemaakt
- Kweek wordt dezelfde dag ingezet op laboratorium microbiologie

- **Consultatiebureau bezoek 11 maanden**
  - Ouders sturen dagboekje en inentingskaart na 3e vaccinatie op naar het onderzoekscentrum. In geval van onduidelijkheden/problemen wordt contact opgenomen met de ouders
  -

- Huisbezoek 3, leeftijd 12 maanden zuigeling
  - Vragenlijst zuigeling invullen
  - Vragenlijst met betrekking tot een van de ouders invullen
  - Vragenlijst met betrekking tot aanwezige broer/zus invullen
  - Transnasale nasopharyngeale kweek bij zuigeling, broer/zus, ouder worden afgenomen
  - Oropharyngeale kweek wordt afgenomen bij ouder
  - Bloedafname zuigeling door venapunctie (1 : 4 deelnemende kinderen groep I en II en 1 : 10 kinderen van de controle groep)
  - Indien venapunctie thuis niet mogelijk of ongewenst, wordt een afspraak gemaakt op polikliniek van het Spaarne Ziekenhuis voor bloedafname door ervaren kinderarts (Dr. R. Veenhoven)
  - Afgeven bloed en kweken dezelfde dag op laboratoria
  - Afspraak wordt gemaakt voor huisbezoek 18 maanden

- Huisbezoek 4, leeftijd 18 maanden
  - Idem 6 maanden, zonder dagboekje postvaccinatie
  - Afspraak voor huisbezoek 24 maanden wordt gemaakt

- Huisbezoek 5, leeftijd 24 maanden
  - Idem 12 maanden

    Tevens
  - Prevenar® vaccinatie 0.5 ml i.m. bij kinderen in groep 3 door onderzoeksarts of verpleegkundige
  - De onderzoeksarts/verpleegkundige vult het onderzoeksformulier/ inentingskaart Prevenar® in met het batchnummer, verloopdatum, datum van vaccinatie en een handtekening. Aangegeven wordt welke vaccinatie is gegeven in welke ledemaat.
  - 15 minuten observatie na de vaccinatie voor eventuele directe lokale of systemische reacties met betrekking tot de gegeven vaccinatie
  - aftekenen op algemene inentingskaart voor de ouders
  - vermelden Prevenar® vaccinatie bij entadministratie

    Tenslotte
  - Er wordt nagegaan of alle formulieren aanwezig zijn en de studie wordt afgesloten

Afwijkingen van de studieprocedure moeten worden gemeld aan de sponsor (UMC) en subsidieverlener (NVI) en moeten zorgvuldig worden gemeld en beschreven in het CRF.

STUDIESCHEMA

Leeftijd baby	Onderzoek	Tijdsbeslag ouders
2-4 weken<br><br><br>Informatiekaart bij MINOES terugsturen	Ontvangst **informatiefolder**, terugsturen **antwoordkaart**<br><br>**Telefonisch contact**, toesturen vragenlijst en toestemmingsformulier. Na 7 dagen opnieuw telefonisch contact voor het maken van afspraak eerste huisbezoek vóór dat de baby 2 maanden oud is	Samen 40 minuten
4-7 weken	**Huisbezoek:** vragenlijst doornemen, toestemmingsformulier tekenen, eerste neuswatje bij de baby. Loten welke inentingen de baby gaat krijgen	30-45 minuten
2 maanden	**Consultatiebureau**: afhankelijk van de groep waarin de baby is ingedeeld **Prevenar®** inenting tegelijk met DaKTP-Hib (en eventueel hepatitis B) prik. **Dagboekje** gedurende een week bijhouden en gedurende die dagen de temperatuur meten.	Dagboekje invullen en temperatuur meten kost 5-10 minuten per dag
4 maanden	**Consultatiebureau:** afhankelijk van de groep waarin de baby is ingedeeld **Prevenar®** inenting tegelijk met DaKTP-Hib (en eventueel hepatitis B) prik. **Dagboekje** gedurende een week bijhouden en temperatuur meten.	Dagboekje invullen en temperatuur meten kost 5-10 minuten per dag
6 maanden	**Huisbezoek:** vragenlijst doornemen en neuswatje bij de baby	30 minuten
11 maanden	**Consultatiebureau:** afhankelijk van de groep waarin de baby is ingedeeld **Prevenar®** inenting tegelijk met DaKTP-Hib (en eventueel hepatitis B) prik. **Dagboekje** gedurende een week bijhouden en die dagen de temperatuur meten.	Dagboekje invullen en temperatuur meten kost 5-10 minuten per dag
12 maanden	**Huisbezoek:** vragenlijst en neuswatje bij de baby, één van de ouders en een broertje of zusje. Keelwatje bij één van de ouders. Bij een deel van de baby's een bloedafname.	45 minuten
18 maanden	**Huisbezoek:** vragenlijst doornemen en neuswatje bij de baby	30 minuten
24 maanden	**Huisbezoek:** vragenlijst en neuswatje bij de baby, één van de ouders en een broertje of zusje. Keelwatje bij één van de ouders.Bij een deel van de baby's bloedafname.<br>**Afsluiten onderzoek** en **Prevenar®** inenting door arts als deze nog niet eerder gegeven zijn.	45 minuten

Voortijdig stoppen met de studie

De subsidiegever heeft het recht om de studie te beëindigen op elk moment als nieuwe gegevens omtrent veiligheid of effectiviteit van het vaccin of het vaccinschema beschikbaar komen tijdens de studie, die vervolgen van de studie ongewenst maken, zelfs in een gecontroleerde situatie. De Ethische Commissie zal worden geïnformeerd over een dergelijke beslissing.

De studie kan worden beëindigd

- als de onderzoeksmedewerker in een situatie komt die verder voortzetting van de studie belemmert en de onderzoeksmedewerker niet kan worden vervangen en geen andere oplossing kan worden gevonden
- als er herhaalde onacceptabele afwijkingen van het onderzoeksprotocol plaatsvinden

Selectie en terugtrekken van studieparticipanten

Inclusiecriteria

- Participanten toelatingscriteria

Alle pasgeborenen die in aanmerking komen voor vaccinaties volgens de richtlijnen van het RVP.

Zuigelingen moeten voldoen aan de volgende criteria

- Goede gezondheid volgens de onderzoeksmedewerker
- De wil en mogelijkheid om het onderzoek volgens de procedure te doorlopen
- Getekend zijn van informed consent
- Leeftijd jonger dan 12 weken en de eerste vaccinatie op leeftijd 2 maanden moet nog plaatsvinden

Exclusie criteria

- Tijdelijke exclusiecriteria

Ziek zijn met matige of hoge koorts (temperatuur > 38.5°C). De vaccinatie wordt uitgesteld tot de ziektesymptomen en verhoogde temperatuur zijn geweken.

- Exclusie criteria

Elk van een van de volgende criteria sluit deelname aan het onderzoek uit

- Aanwezigheid van een ernstige ziekte die medische zorg vereist die kan interfereren met de resultaten van de studie
- Bekende of vermoede allergie/overgevoeligheid tegen een van de vaccinbestanddelen (anamnestisch, alert zijn als het kind medische klachten heeft gehad na vaccinaties met Prevenar®)
- Bekende of vermoede afweerstoornis
- Tevoren toegediend zijn van plasmaproducten (inclusief immuunglobulinen)
- Tevoren reeds gevaccineerd zijn, anders dan met hepatitis B vaccinatie
- Bloedingstoornissen

Terugtrekken uit de studie

Zuigelingen mogen elk moment uit de studie worden teruggetrokken door het informed consent terug te trekken.

De artsonderzoekers kunnen deelname aan de studie voortijdig beëindigen indien

- Er adverse events optreden na de vaccinatie die verdere giften gecontraïndiceerd maken
- Intercurrente ziekte zich tijdens de studie voordoet die de studie resultaten beïnvloedt of gezondheidsrisico voor de participant kunnen betekent bij vervolg vaccinaties. De Principal Clinical Investigator zal deze gevallen individueel evalueren
- De participant is lost-to-follow-up, in geval van verhuizen, communicatieproblemen, etc
- Overtredingen van het protocol/ de studieprocedures

In geval van vroegtijdig stoppen, zal het CRF tot dat moment worden gecompleteerd. De ouders worden gevraagd het dagboek en de vragenlijsten te completeren.

**Voortijdig studie stoppen**	**afwerken**	**Bij voorkeur**
Voor eerste vaccinatie en/of kweek	Vul stop formulier in van CRF	
Na 1e of 2e vaccinatie op 2 en 4 maanden	Vul stopformulier in van CRF	Werk de vragenlijst van 6 maanden af.<br>Werk dagboekjes af.<br>Neuskweek van de zuigeling.
Na 11 maanden	Vul stopformulier in van CRF	Werk vragenlijsten van 12 (18 of 24 maanden) af.<br>Werk dagboekje 11 maanden af.<br>Neuskweek van de zuigeling.

Er zullen geen vervangende kinderen worden opgenomen in de studie.

Fasering van het onderzoek

- Januari 2005: indienenSTEG. Overleg entadministraties, Stichtingen Thuiszorg. Informeren huisartsen, verloskundigen, poliklinieken verloskunde, consultatiebureau-artsen en medewerkers.
- Mei 2005: Zodra goedkeuring van deSTEG, drukken folders, vragenlijsten, informed consent en brieven.

Afhankelijk van definitieve goedkeuring (STEG) beoogde startdatum:

- 23 juni 2005: Start werving via de entadministratie.
- September/Oktober 2006: beoogde interim-analyse
- Beoogde einddatum follow-up december 2007
- 1 januari 2008 – 1 april 2009: data-analyse, rapportage en verslaglegging via publicaties

Vaccingegevens

- Product

- Prevenar®

Elke dosis van 0,5 ml voor i.m. injectie bevat 2 microgram van het polysaccharide serotype 4, 9V, 14, 19F en 23F, 2 microgram van het oligosaccharide van het serotype 18C en 4 microgram van het polysaccharide serotype 6B. Het is geconjugeerd aan het CRM_{197} dragereiwit en geabsorbeerd aan aluminiumfosfaat (0,5 mg). De andere ingrediënten zijn natriumchloride en water voor injecties.

- Verpakking en labelen

Prevenar wordt door het NVI via de entadministraties verstrekt aan de betreffende consultatiebureaus in de huidige verpakkingsampullen à 0,5 ml per vaccin, zoals geleverd door de fabrikant Wyeth. Het vaccin moet lege artis worden opgetrokken in een spuit voor i.m. injectie.

- Dosering en toediening

De ampul moet voor toediening worden geschud, de oplossing moet helder zijn. Voor optrekken uit de ampul met de spuit moet er op de ampul worden getikt zodat alle vaccin het laagste punt van de ampul bereikt en kan worden opgetrokken. Aseptische technieken moeten lege artis worden toegepast voor elke dosis. De suspensie moet direct diep intramusculair worden toegediend, bij voorkeur in de bovendij (musculus quadriceps femoris) rechts of links maar in een andere ledemaat dan waar de DaKTP-Hib wordt toegediend. Bij een eventuele Hepatitis B vaccinatie vindt Prevenar® toediening plaats in een bovenarmpje.

Ingevuld worden op het onderzoeksformulier

- UTN
- Batchnummer
- Expiratiedatum
- Ledemaat
- Naam, datum en handtekening van degene die heeft gevaccineerd
- Eventuele bijzonderheden

- Voorziening en opslag

De trial vaccins zullen aan de onderzoeksmedewerkers/consultatiebureaus worden verstrekt door het NVI via de entadministraties. De onderzoeker is verantwoordelijk voor correcte opslag en transportcondities bij +2 - + 8 °C naar locatie van vaccinatie. De vaccins worden vervoerd in geïsoleerde koelboxen onder continue temperatuursmonitoring die aantoont dat de temperatuur adequaat blijft. Producten mogen niet bevriezen.

- Vervanging onbruikbare vaccins

Niet bruikbare vaccins, zoals die waarvan de expiratiedatum is verstreken of beschadigde ampullen zullen aan het NVI worden geretourneerd en worden vervangen.

- **Study Product Accountability**

Elk vaccin vervoer moet worden voorzien van een getekend receptuur formulier met informatie over kwantiteit, expiratiedatum en batchnummers van de geleverde vaccins. Het formulier moet gedateerd zijn en getekend door de persoon verantwoordelijk voor het transport en de persoon die het product aanneemt. De onderzoeker is verantwoordelijk voor de accountability op locatie. Een geschreven verklaring moet door de onderzoeker worden verstrekt indien er discrepantie bestaat tussen het geleverde en gebruikte aantal vaccins aan het einde van de studie. Alle ongebruikte vaccins worden geretourneerd aan het NVI aan het einde van de studie.

Veiligheidsmonitoring van vaccinaties

- **Veiligheidsmonitoring**

Zuigelingen en peuters worden de eerste 15 minuten na vaccinatie geobserveerd door de ouder op het consultatiebureau. Een dagboek gedurende 7 dagen dient om alle gezondheidsgegevens vast te leggen van de week aansluitend op de vaccinaties op 2, 4 en 11 maanden.

De lichaamstemperatuur wordt rectaal gemeten op de dag van vaccinatie en daarna 6 dagen aansluitend. De hoogste temperatuur wordt dagelijks genoteerd in het dagboek.

- **Adverse events**

Een adverse event wordt gedefinieerd als elke negatieve medische gebeurtenis bij een deelnemer van het onderzoek die een farmaceutisch product toegediend heeft gekregen maar wat niet noodzakelijk verband hoeft te houden met deze toediening. Het kan elk ongunstig, afwijkend en onbedoelde bevinding, ziekte of symptoom zijn, in tijd geassocieerd met het toedienen van het pneumokokken vaccin Prevenar®.

Een serieus adverse event wordt gedefinieerd als elke onverwachte wijziging in de medische situatie die

- leidt tot de dood
- levensbedreigend is
- opname in een ziekenhuis noodzakelijk maakt of een bestaande opname verlengd
- resulteert in blijvende nadelige gevolgen of handicaps

Voor de relatie met gegeven vaccinaties worden de volgende classificaties gebruikt

- Onwaarschijnlijk. Er is geen medische evidentie dat suggereert dat het adverse event' is gerelateerd aan de vaccinatie of er is een andere, meer plausibele verklaring
- Mogelijk. Er is medisch gezien een mogelijke verklaring dat het event is gerelateerd aan de vaccinaties. Echter, andere medische verklaringen kunnen niet worden uitgesloten als oorzakelijk verband.

- Waarschijnlijk. Er is sterke medische verdenking op een causaal verband tussen de gegeven vaccinatie en het optreden van het adverse event. Er bestaan geen andere mogelijke medische verklaringen voor het adverse event.

Elk SAE wordt gemeld bij het onderzoekscentrum en zorvuldig geregistreerd en beoordeeld door de data safety monitoring commissie.
Een onafhankelijke data safelty monitor commissie zal worden aangesteld om met intervallen de voortgang van de studie, de veiligheidsmonitoring en data en het volgen van de studieprocedures te evalueren. Dit team zal bestaan uit een kinderarts (Dr T. Wolfs), een consultatiebureau arts (mevr K. Stam), een epidemioloog (Dr C. Uiterwaal) en twee senior wetenschappelijk medewerkers van klinische studies bij het NVI (Dr N. Rots, R. Burgmeijer). De commissie zal tenminste elk half jaar gedurende de studie bijeenkomen en zodra alle data beschikbaar zijn. In geval van een ernstig onverwacht serieus adverse event (SUSAR) dat mogelijk gerelateerd is aan de vaccinatietoediening, zal onverwijld het event worden doorgegeven aan de vaccinleverancier Wyeth en aan het NVI en altijd binnen 14 dagen aan het CBG met een CIOMS formulier. De onderzoeksartsen (L.sanders, R.Veenhoven, E. van Gils) kunnen beoordelen of een SAE een SUSAR is. Bij twijfel wordt de data safety monitoring commissie geraadpleegd.

- Bijwerkingen Prevenar®

Bijwerkingen die vaak (meer dan 1 : 10 patiënten) optreden zijn verminderde eetlust, braken, diarree, pijn, gevoeligheid, roodheid of zwelling en verharding van de injectieplaats; koorts van 38 °C of meer, prikkelbaarheid, slaperigheid of onrustige slaap. Normaal (minder dan 1 : 10 patiënten) komen voor roodheid of zwelling en verharding van de injectieplaats groter dan 2, 4 cm, gevoeligheid van de injectieplaats die de beweging van de ledemaat beperkt, koorts van 39 °C of meer. Soms (minder dan 1 : 100 patiënten) bultjes/uitslag (urticaria). Zelden (minder dan 1 : 1000 patiënten) stuipen, koortsstuipen; overgevoeligheidsreacties op de injectieplaats zoals uitslag/ urticaria of bultjes en jeuk; hypotoon-hyporesponsieve episode; ernstige allergische/overgevoeligheidsreacties welke zwelling van de lippen, het gezicht kan omvatten, moeite met ademhalen en/of shock. Zeer zelden (minder dan 1 : 10.000) vergroten van de lymfeklieren van het gebied rond de injectieplaats, erythema multiforme.
Antipyretische medicatie wordt aanbevolen bij gelijktijdig vaccineren met whole cell pertussis vaccin, bij kinderen met epileptische stoornissen of kinderen bekend met koortsstuipen. Voorts is het gerechtvaardigd bij koorts meer dan 39 °C na vaccinatie.

- Verwachte adverse events

- Lokale symptomen:

- Roodheid en zwelling; score in millimeters, hoogste score telt
- Verminderd gebruik van de geïnjecteerde extremiteit
- Pijn

- Systemische reacties

- Koorts; rectaal gemeten

- Sufheid
- Ongewoon of ontroostbaar huilen (uren)
- Minder drinken/eetlust
- Abnormaal/onrustig slaappatroon
- Diarree
- Huidafwijkingen
- Collaps

Naar deze symptomen wordt in het dagboekje gevraagd.

- Documenteren adverse events

Alle adverse events of complicaties aangegeven door de ouders/verzorgers of waargenomen door de onderzoeksmedewerker moeten worden gedocumenteerd in het CRF. Serious adverse events worden bovendien op SAE-formulieren apart gerapporteerd. De volgende categorieën van ernst voor de gedefinieerde adverse events worden gebruikt:

**Lokale reacties**	**Intensiteit**	**Diameter van lokale reactie**
Roodheid en zwelling	Mild	< 10 mm
	matig	10-29 mm
	ernstig	30 mm of meer

**Systemische reacties**		
koorts	1	37,5-38,4 °C
	2	38,5-39,4 °C
	3	39,5-40,4 °C
	4	40,5 °C
Ongewoon of ontroostbaar huilen	mild	1 uur
	matig	1-3 uur
	ernstig	3 uur
Suf zijn	ernstig	3 uur
Minder actief	Aan- of afwezig	
Minder drinken/eten	Aan- of afwezig	
Spugen/braken	Aan- of afwezig	
Diarree	Aan- of afwezig	

Een SAE moet zo spoedig mogelijk aan het onderzoekscentrum, de sponsor (UMC) en de subsidieverlener (NVI) en de studiecoördinatoren (R.H. Veenhoven, EAM Sanders, E van Gils)

worden gemeld, maar altijd binnen 24 uur (telefoonnummer: 023-890 9070). Details van het SAE worden verstrekt op het SAE formulier en gefaxt door de onderzoeker naar de producent van het vaccin (Wyeth) en de subsidiegever (NVI). Vervolg informatie moet worden verstrekt zodra dit beschikbaar is.

Afnameprocedure en microbiologie van nasopharyngeaal dragerschap

- **Afname procedure en inzetten transnasale nasopharyngeale kweken**

Door speciaal getrainde (GCP) research nurses, onderzoeksassistenten of artsen wordt met een steriel flexibel wattenstokje transnasaal de nasopharynx uitgestreken. De flexibele wattendrager (Copan Italia, Brescia, Italy) wordt via het neusgat ingebracht en zachtjes via de neusbodem opgevoerd tot de weerstand van de achterste pharynxwand wordt gevoeld. Dan wordt voorzichtig uitgestreken (1-2 seconden zachtjes bewegen) en vervolgens het watje teruggetrokken en geplaatst in Stuart transportmedium bij kamertemperatuur. De kweek wordt binnen 24 uur uitgeplaat op selectieve platen op het Streeklaboratorium voor Microbiologie te Haarlem.

**Selectieve plaat**	**Type bacterie**
Bloedagar met 5 mg/l gentamicine	*S. pneumoniae*
Bloedagar	*S. pneumoniae, S. aureus, M. catarrhalis*
Chocolade agar	*H. influenzae, M. catarrhalis*
Haemophilus chocolade	*H. influenzae*
Bordet-gengou (+PCR)	*B. pertussis, B. parapertussis*
Mannitolzoutagarplaat	*S. Aureus*
Pikeplaat	*ß haemolytische streptokok (A, C, G)*

De resultaten worden semikwantitatief bepaald (+, ++, +++, ++++). In geval van groei S. pneumoniae zal serotypering plaatsvinden met de kapselzwellingsmethode (Quellung reactie) met commercieel verkrijgbare antisera (Statens Serum Institute, Copenhagen, Denmark). Het kweekmateriaal wordt 15 jaar bewaard en dan vernietigd.

- **Afname procedure en inzetten oropharyngeale kweken**

Door speciaal getrainde (GCP) research nurses, onderzoeksassistenten of artsen wordt een steriel wattenstaafje via de mondholte onder direct zicht naar binnen gebracht en de posterieure pharynxwand en (éénzijdig) tonsilnis uitgestreken (1-2 seconden zachtjes bewegen). Vervolgens wordt de wattendrager teruggetrokken en geplaatst in Stuart transportmedium bij kamertemperatuur. De kweek wordt binnen 24 uur uitgeplaat op selectieve platen voor pneumokokken op het Streeklaboratorium voor Microbiologie te Haarlem.

Pneumokokken infecties

Invasieve pneumokokkenmeningitis oorzaken en serotypen worden vastgelegd via het reeds bestaande landelijke registratie systeem via (Nationaal Referentielaboratorium voor Bacteriële Meningitis, Amsterdam). Meningitis- isolaten worden bewaard voor onderzoek.

Gegevens met betrekking tot artsbezoeken, oorontstekingen, luchtweginfecties, antibioticagebruik en evt. ziekenhuisopnamen worden na toestemming van de ouders via de huisarts of specialist opgevraagd.

Immunologische bepalingen

Immunogeniciteits vergelijkingen van 2 versus 3 Prevenar vaccinaties worden verricht op leeftijd 12 en 24 maanden.[1] De hoogte van IgG antipneumokokken antistoftiters tegen de 7 vaccin kapsel polysacchariden wordt bepaald met een ELISA volgens internationale richtlijnen.[2,3] Aviditeitsbepalingen dienen als aanvullende merker voor immunologisch geheugen.[1,4] Opsonophagocytosis wordt daarnaast internationaal aanbevolen als deel van de evaluatie van huidige en toekomstige pneumokokken vaccins.[1,5,6]

Voor het huidige onderzoek meten wij in duplo de IgG titers (ELISA) tegen de 7 pneumokokken serotypen opgenomen in het vaccin, de kruisreagerende, veel voorkomende serotypen 6A en 19A en serotypen welke waarschijnlijk toenemen na vaccinaties met Prevenar (7, 11, 15, 16). Hiervoor reserveren wij 400 microliter serum. Aanvullend verrichten wij eveneens opsonophagocytosis (vaccinserotypen, 6A en 19A, 11 en 15) en aviditeitsmetingen waarvoor eveneens 400 microliter serum nodig is.

Indien onvoldoende serum beschikbaar is voor alle bepalingen zullen wij allereerst de ELISA verrichten en afhankelijk van de beschikbare hoeveelheid serum vervolgens voorrang geven aan de aviditeit boven de opsonophagocytosis.

1. Jodar J, Butler J, Carlone G, Dagan R, Goldblatt D et al. Serological criteria for evaluation and licensure of new pneumococcal conjujate vaccine formulations for use in infants. Vaccine 2003; 21:3265-3272
2. Concepcion NF, Frasch CE. Pneumococcal type 22F polysaccharide absorption improves the specificity of a pneumococcal-polysaccharide enzyme-linked immunosorbent assay. Clin and Diagnostic Laboratory Immunology 2001;8:266-727
3. Quataert SA, Rittenhouse-Olson K, Kirch CS, Hu B, Secor S, et al. Assignment of weight-bases antibody units for 13 serotypes to a human antipneumococcal standard reference serum, lot 89-S(F). Clin and Diagnostic Laboratory Immunology 2004;11:1064-1069
4. Kilpi et al. Protective efficacy of a second pneumococcal conjugate vaccine against pneumococcal acute otitis media in infants and children: randomized, controlled trial of a 7-valent pneumococcal polysaccharide-meningococcal outer membrane protein complex conjugate vaccine in 1666 children. Clin Infect Dis. 2003 Nov 1;37(9):1155-64.
5. Romero-Steiner S, Frasch C, Concepcion N, Goldblatt D, Kayghty H et al. Multilaboratory Evaluation of a viability assay for measurement of opsonophagocytic antibodies specific to the capsular polysaccharides of Streptococcus pneumoniae. Clin and Diagnostic Laboratory Immunology 2003;10:1019-1024
6. Hu BT, Xinhong Y, Jones TR, Kirch C, Harris S et al. Approach to validating an opsonophagocytic assay for Streptococus pneumoniae. Clin and Diagnostic Laboratory Immunology 2005;12: 278-295

Statische analyse

De onafhankelijke Data Monitor Commissie zal de verzamelde data van de studie evalueren en aanbevelen welke data in de analyses mogen worden meegenomen.

- Primaire eindpunt

- Reductie van pneumokokken VT dragerschap door zuigelingen na 2 en 3 Prevenar® vaccinaties in vergelijking met een ongevaccineerde controle groep

- Secundaire doelstellingen

- Reductie van pneumokokken dragerschap in het gezin (ouder, broertje/zusje)
- Onderzoeken en voorspellen van effecten van ‘herd-immunity’
- Onderzoeken welke vaccintype (VT) en non-vaccintype (NVT) pneumokokken in Nederland circuleren voorafgaand aan Prevenar® invoering
- Onderzoeken welke NVT pneumokokken opkomen na pneumokokken conjugaatvaccinaties
- Volgen van de relatie NP dragerschap en invasieve pneumokokkenziekten (IPD)
- Antistoftiters bepalen na 2 respectievelijk 3 Prevenar® vaccinaties op 12 en 24 maanden
- Evalueren van eventuele reductie van community-acquired acute otitis media en lage luchtweginfecties als pneumonie na pneumokokken conjugaatvaccinatie
- Evaluatie van andere koloniserende species als *S. Aureus, B. pertussis*
- Monitoren veiligheid vaccin

- Bepaling van de groepsgrootte

Voor de berekening van de benodigde groepsgrootte gaan we uit van het primaire eindpunt VT pneumokokkendragerschap (zie boven). De power van de studie wordt met name bepaald door het kleinste te verwachten effect, na 2 en 3 Prevenar® vaccinaties. Verder is het gebruikelijk een statistisch significantie niveau (α) aan te houden van 5% en een power ($1-\beta$) van 80%. Om een statistisch significant verschil te kunnen aantonen in de reductie van VT pneumokokken dragerschap bij 2 en 3 Prevenar® vaccinaties van 35% naar 25% van de VT pneumokokkenisolaten uit de nasopharynx, moeten er bij een alfa van 5% en beta van 80%, 300 kinderen in iedere groep worden ingesloten (berekend met EPI-INFO versie 6). Rekening houdend met een uitval van 10% betekent dit een inclusie van 1000 zuigelingen in de studie.

Participantendiagram

- Aanschrijven ouders van nieuwgeborenen in Haarlem e.o. (n=9.000)
- Telefonische informatie na terugsturen antwoordkaart (n=)
- Toesturen informatie en informed consent (n=)

Toestemming informed consent (n=…)
Randomisatie in 3 groepen

Groep I	Groep II	Groep III
Prevenar op 2 en 4 mnd (n=)<br>Afgevallen (n=)	Prevenar op 2, 4 en 11 mnd (n=)<br>Afgevallen (n=)	Prevenar 24 mnd (n=)<br>Afgevallen (n=)
Teruggetrokken (n=)<br>Interventie volledig (n=)<br>Lost-to-follow-up (n=)<br>Andere redenen (n=)	Teruggetrokken (n=)<br>Interventie volledig (n=)<br>Lost-to-follow-up (n=)<br>Andere redenen (n=)	Teruggetrokken (n=)<br>Interventie volledig (n=)<br>Lost-to-follow-up (n=)<br>Andere redenen (n=)
Trial afgemaakt (n=)	Trial afgemaakt (n=)	Trial afgemaakt (n=)

- Studiepopulatie

De primaire analyse populatie zullen alle kinderen zijn met minimaal één afgenomen nasopharyngeale kweek. Safety data analyses worden uitgevoerd op alle gevaccineerde kinderen die minimaal een Prevenar® vaccinatie hebben gehad.

- Demografische analyse

Beschrijvende statistiek en frequentietabellen zullen worden gepresenteerd per interventie groep.

- Data analyse

De data zullen geanalyseerd worden volgens het intention-to-treat principe. Descriptieve statistiek zoals frequentieverdelingen voor dichotome/nominale variabelen en gemiddelde (SD)/mediaan (P25,P75) zullen gebruikt worden om de verdeling van baseline kenmerken in de drie onderzoeksgroepen te beschrijven. Primair zullen relatieve risico's en bijbehorende 95% -betrouwbaarheidsintervallen berekend worden op het optreden van VT pneumokokkendragerschap (en de secundaire eindpunten) op basis van de cumulatieve incidentie na 24 maanden. Hierbij geldt de controlegroep als referentie en de twee overige groepen als index groep. Met behulp van de chi-kwadraat toets zullen de verschillen in proporties en met de t-toets of verdelingsvrije variant zullen verschillen in gemiddelden of medianen statistisch worden getoetst. Tevens zal een multilevel model (GEE) worden ontwikkeld om zowel tijdsgerelateerde effecten als binnen-persoons effecten tengevolge van herhaalde metingen te kunnen schatten.

- **Interim analyse**

6 maanden nadat alle kinderen het volledige vaccinatieschema van 2, 4 en 11 maanden hebben doorlopen zal een interim analyse worden verricht op de uitkomsten van de nasopharyngeale kweken en de antistoftiters op 12 maanden.

Methodologie randomisatie in groepen.

Het betreft een niet-geblindeerd onderzoek. Een gecomputeriseerd randomisatieschema zal worden gebruikt om de participanten aselect toe te wijzen aan een van de drie onderzoeksgroepen. (verwijderd Opeenvolgende nummers zullen worden toegekend aan de participanten door de onderzoeker. Het datamanagement zal het toegewezen bijbehorende allocatienummer met beschrijving van de onderzoeksgroep toesturen aan de participant. Hetzelfde unieke identificatienummer zal gehanteerd worden gedurende de hele studie.)

Toegang tot de code

Directe toegang tot de bron, data documenten, kwaliteitscontrole en kwaliteitsverzekering.

Door het protocol te tekenen geeft de onderzoeker permissie aan het personeel van de subsidiegever, aan geautoriseerde medewerkers van het NVI, aan de bevoegde autoriteiten en/of de ethische commissieleden on-site trial-related monitoring te verrichten, evenals on-site audits, IEC review en inspectie van de procedures, met directe toegang tot brondata en documenten. Audits houden onder meer in het reviewen van bron documenten ter verificatie van het adequaat en zorgvuldig vastleggen van case-report formulieren, documentatie die verricht moet worden en controles of vaccin accountability**.**

Medische Ethiek

- **Onafhankelijke ethische commissie**

Alvorens de trial te starten moet er schriftelijke en van een datum voorziene toestemming zijn van een onafhankelijke ethische commissie (Independent Ethics Committee, IEC) voor het trial protocol, de tekst van het informed consent en overige schriftelijke informatie die aan ouders wordt verstrekt. Veranderingen in het studie protocol moeten worden beschreven in een amendement van het protocol. Schriftelijke toestemming van de IEC is vereist voor de wijziging mag worden geïmplementeerd.

De onderzoeker moet alle correspondentie met de IEC bewaren. Bovendien moet de onderzoeker alle informatie over ernstige onverwachte gebeurtenissen/serieus adverse event melden aan de IEC. Jaarlijks moet een rapport worden verstrekt over de voortgang van de studie aan de IEC.

- **Informed Consent**

De onderzoeker of een bevoegde medewerker van het onderzoek moet de ouders volledig informeren over alle relevante aspecten van de studie met betrekking tot de aard, het perspectief en de mogelijke consequenties van deelname aan de studie door zowel mondelinge als schriftelijke informatie. Alle geschreven informatie moet worden goedgekeurd door de IEC alvorens het mag worden gebruikt.

Na alle informatie te hebben gekregen en te begrijpen, moeten beide ouders danwel de wettelijke vertegenwoordiger toestemming geven voor deelname aan het onderzoek door persoonlijk het informed consent formulier te tekenen en van datum te voorzien. Daarnaast moet de bevoegde onderzoeksmedewerker die de informed consent discussie heeft gevoerd eveneens het informed consent persoonlijk tekenen en van datum voorzien. Een kopie van het informed consent formulier wordt aan de ouders/wettelijk vertegenwoordiger verstrekt. Het originele formulier wordt bewaard door de onderzoeker.

De onderzoeker gaat geen acties ondernemen die vereist zijn voor de studie totdat een valide informed consent is verkregen.

- **Regulatoire overwegingen**

De klinische studie zal worden uitgevoerd conform de geldende regels van Good Clinical Practice, zoals vastgelegd door de Committee for Proprietary Medical Products (CPMP) van de Europese Unie en de International Committee on Harmonisation (ICH) in " Note for Guidance on Good Clinical Practice, document CPMP/ICH/135/95" effectief sinds 17 januari 1997, het Clinical Trial Directive 2001/20/EC, The World Medical Declaration of Helsinki en de bijbehorende amendementen in gebruik na 1964 en voldoend aan de nationale regulatoire vereisten.

- **Verantwoordelijkheid van de onderzoeker**

De onderzoeker moet de studie uitvoeren in overeenstemming met dit protocol, GCP en andere lokale wettelijke vereisten en regels.
De onderzoeker moet het protocol tekenen en verzekeren dat alle personen die assisteren bij de studie adequaat zijn geïnformeerd over het protocol, protocolwijzigingen, vaccinaties en studie-gerelateerde verplichtingen en functies.

De onderzoeker (investigator) moet een lijst bijhouden van alle sub-investigators en ander gekwalificeerde personen aan wie hij of zij belangrijke studie gerelateerde verplichtingen/taken heeft gedelegeerd met een schriftelijk vastleggen van de overgedragen verplichtingen/taken.

Vertrouwelijkheid, data management en bijhouden van registratie

- **Uniek Trial Number**

Elk kind waarvan een getekend, schriftelijk informed consent beschikbaar is krijgt een uniek trial nummer toegewezen (UTN). Dit UTN wordt gebruikt om het kind te identificeren in de studie en wordt vermeld op alle studiedocumenten. Hetzelfde geldt voor ouders en het participerende broertje of zusje. De naam van het kind en gezinsleden worden niet aan de sponsor verstrekt. De Clinical Investigator verzekert dat de anonimiteit van het kind en gezinsleden zijn is gewaarborgd. Een apart logboek van UTN nummers, namen en adressen van deelnemers wordt bijgehouden. Kinderen welke uit de studie worden teruggetrokken houden hun UTN.

- **Data verwerking**

Alle data vereist volgens het protocol die tijdens de studie worden verzameld, moeten worden vastgelegd op Case Report Forms (CRFs) door de onderzoeker of een geautoriseerde onderzoeksmedewerker of door de ouders/verzorgers in het dagboek. Geen persoonlijke identificatie komt voor op CRF formulieren of dagboek, met uitzondering van de geboortedatum, initialen en geslacht. De CRFs en de dagboeken zijn het bezit van de sponsor van de studie. De originele data worden overhandigd aan de sponsor; kopieën van de CRFs worden bewaard door de onderzoeker. Studie data en bevindingen opgeslagen op een computer zullen worden opgeslagen in overeenstemming met lokale data protectie regelgeving.

- **Studie monitoring**

Een vertegenwoordiger van de sponsor zal de studie monitoren gedurende de diverse studie perioden. De monitor zal het volgende in acht nemen:

- Voorzieningen en procedures van de trial
- Aanwezigheid van schriftelijke toestemming van de IEC

- Werken in overeenstemming met het protocol
- Aanwezigheid van persoonlijk getekende en gedateerde informed consent formulieren
- Zorgvuldigheid en volledig zijn van vastgelegde onderzoeksdata op de CRFs
- Consistentie tussen brondata en data genoteerd op het CRF
- Rapporteren van ‘serieus adverse events’
- Verantwoordelijkheid en maatregelen bij ‘serieus adverse events’
- Vaccin opslag en transport
- Overeenstemming met het studie schema
- Beschikbaarheid van de onderzoeker bij consultatie in geval van incidenten
- Aanwezigheid van een up-to-date ‘trial site file’

- **Bewaren van studiegegevens door onderzoeker**

Essentiële documenten, in overeenstemming met GCP, en bron documenten moeten worden bewaard op studielocatie voor een periode van 15 jaar nadat de laatste deelnemer de studie heeft afgesloten.
De sponsor mag langer bewaren van data vragen. De onderzoeker moet om die reden schriftelijk toestemming hebben van de sponsors alvorens datarecords te mogen vernietigen.

- **Financiering van het onderzoek**

Financiering wordt bepaald in de subsidieovereenkomst (research agreement) met de onderzoeker. Gegevens over de verzekering worden gegeven in de deelnemers informatie/informed consent documenten. De verzekering wordt afgesloten door de subsidiegever (VWS, vertegenwoordigd door het NVI).

- **Publicatie beleid**

De onderzoeker zal in overeenkoming met het NVI een definitief rapport opstellen zodra als mogelijk na het sluiten van de studie. Dit laatste rapport wat moet zijn goedgekeurd door het NVI, welke toestemming afhankelijk zal zijn van de presentatie van de onderzoeksresultaten met het doel registratie voor een 2-doses schema Prevenar in het RVP te ondersteunen. Het publicatiebeleid is vastgelegd in de subsidieovereenkomst van het NVI met het UMC.

- **Voortijdig beëindigen van de studie**

Indien de studie voortijdig wordt gestaakt, moet de IEC hiervan op de hoogte worden gebracht en moet alle studiemateriaal aan het UMC worden teruggegeven zodra dit volledig is afgemaakt.

Bijlagen

1. Informatiefolder zoals verstuurd door entadministratie
2. Informed consent

Bijlage 1
Tekst MINOES informatiefolder zoals verstuurd door entadministratie (wervingsfolder)

Geachte ouders en verzorgers,

U krijgt van de entadministratie straks informatie over de vaccinaties die uw kindje gaat krijgen via het Rijksvaccinatieprogramma. Mogelijk wordt in de toekomst aan dit schema een nieuwe belangrijke vaccinatie toegevoegd: de pneumokokkenprik Prevenar®. Deze inenting beschermt tegen hersenvliesontsteking veroorzaakt door pneumokokken. De pneumokokkenprik zal driemaal worden gegeven op de leeftijd van 2, 4 en 11 maanden, tegelijk met de DaKTP-Hib vaccinaties. Het Ministerie voor Volksgezondheid, Welzijn en Sport heeft echter subsidie gegeven voor onderzoek naar de vraag of het vaccinatieschema eenvoudiger kan. Omdat u in de regio woont waar dit onderzoek plaats vindt, nodigen we u uit voor deelname aan MINOES: het "**Mi**nder prikken **N**eus **O**nderzoek; **E**ffect bij baby en **S**amenleving". Met de resultaten kan het vaccinatieschema in de toekomst mogelijk minder belastend worden gemaakt voor alle baby's. Uw kindje kan vast de pneumokokkeninenting krijgen alvorens dit landelijk wordt ingevoerd.

Het MINOES onderzoek: twee- of driemaal een pneumokokkenprik?
Er zijn aanwijzingen dat twee pneumokokkeninentingen op de leeftijd van 2 en 4 maanden uw kindje evengoed beschermen tegen hersenvliesontsteking als drie. Of dit echt zo is, willen wij onderzoeken in het zogenaamde MINOES onderzoek. Een goede manier hiervoor is kijken hoe vaak vóór en ná twee of drie keer inenten de pneumokok in de neus-keelholte van de baby wordt gevonden. Dit kan door met een watje achter in de neus wat slijm af te nemen en dit te kweken.
We weten dat door inenting met het pneumokokkenvaccin niet alleen bij de ingeënte baby maar ook bij zijn/haar ouders en broers en zussen de pneumokokken waartegen je vaccineert minder vaak voorkomen in de neus. Baby's zijn namelijk een belangrijke bron voor het verspreiden van pneumokokken binnen het gezin en daarbuiten. Daarom willen we ook graag, zo mogelijk, bij één van de broers of zussen met neusuitstrijkjes en bij één van de ouders met een neus- en keeluitstrijkje onderzoeken wat het effect is van twee- of driemaal inenten van de baby.
Door de kweken op pneumokokken van de neuswatten te vergelijken tussen baby's die twee of drie keer zijn ingeënt met baby's die niet zijn gevaccineerd met de pneumokokkenprik en de uitstrijkjes van hun gezinsleden te vergelijken, kunnen wij vaststellen of het voldoende is in de toekomst twee in plaats van drie pneumokokkenprikken te geven aan alle baby's.

Ziekte door pneumokokken.
Iedereen heeft van tijd tot tijd pneumokokken in de neus-keelholte. Ze worden van mens op mens overgedragen en men wordt er meestal niet ziek van. Zuigelingen en peuters dragen veel vaker

pneumokokken in de neus-keelholte en worden er ook vaker ziek van omdat zij minder afweerstoffen hebben. Als dan de weerstand vermindert of een verkoudheid optreedt, kan de pneumokok vanuit de neus-keelholte een oorontsteking of longontsteking geven. Heel zelden komt de pneumokok in de bloedbaan terecht en kan dan een hersenvliesontsteking veroorzaken. In Nederland overlijden nu jaarlijks nog 10-15 zuigelingen aan de gevolgen van deze ernstige infectie.

De pneumokokkenprik Prevenar®

Er zijn wel meer dan 90 soorten pneumokokken. Gelukkig veroorzaakt maar een kleine groep van 7 typen de meeste pneumokokkeninfecties. In het huidige pneumokokkenvaccin (Prevenar®) zijn deze 7 typen opgenomen. In de Verenigde Staten zijn inmiddels miljoenen baby's ingeënt met dit vaccin. Het is een veilig vaccin, gemaakt van deeltjes van de buitenkant (het kapsel) van de pneumokok. Deze deeltjes zijn gekoppeld aan een eiwit. De bijwerkingen van Prevenar zijn vergelijkbaar met de DaKTP-Hib inenting: er kan roodheid, zwelling en pijn ontstaan op de prikplaats en sommige kinderen krijgen koorts. Deze bijwerkingen zijn na 1 tot 3 dagen verdwenen. Heel belangrijk is dat na inenting met het pneumokokkenvaccin de kinderen voor meer dan 95% beschermd zijn tegen hersenvliesontsteking door pneumokokken die in het vaccin zijn opgenomen. De prik beschermt ook maar minder goed tegen longontsteking en een beetje tegen oorontsteking.

Hoe gaat het MINOES onderzoek?

Allereerst vragen we aan u als ouders of verzorgers of u belangstelling heeft om mee te doen met uw baby aan het onderzoek door de portvrije antwoordkaart in te vullen met uw telefoonnummer en vervolgens op te sturen. Zodra wij uw kaart hebben ontvangen, nemen wij telefonisch contact met u op en zullen het onderzoek met u doorspreken. Als u denkt mee te doen, zullen we u uitgebreidere informatie en een toestemmingsformulier (verwijderd: 'en een vragenlijst') toesturen. Leest u deze rustig door. Na een week nemen we opnieuw contact op voor het maken van een afspraak bij u thuis. Tijdens dit huisbezoek spreken we het onderzoek (verwijderd: 'en de vragenlijst') nog eens met u door. Als u akkoord bent, tekenen zowel u als de onderzoeksmedewerker het toestemmingsformulier. Dan kan het MINOES onderzoek voor u en de baby van start gaan.

Door loting wordt uw kindje vervolgens ingedeeld in één van de volgende drie groepen:

1. Een pneumokokkenprik op de leeftijd van 2, 4 en 11 maanden
2. Een pneumokokkenprik op de leeftijd van 2 en 4 maanden
3. Een pneumokokkenprik aan het einde van het onderzoek op de leeftijd van 24 maanden

Het eerste neuswatje bij uw kindje wordt vervolgens afgenomen. Na dit eerste bezoek komen we nog 4 keer bij u thuis voor het afnemen van een neuswatje en het invullen van vragenlijsten als uw kindje 6, 12, 18 en 24 maanden oud is.

Tijdens het huisbezoek op de leeftijd van 12 en 24 maanden nemen we niet alleen een neuswatje af bij uw baby maar ook bij één van de eventuele broertjes of zusjes. Bij één van de ouders nemen we dan een neus- én keelwatje af.

De pneumokokkenprikken op 2, 4 en 11 maanden worden tegelijk met de DaKTP-Hib prik gegeven op het consultatiebureau maar in het andere bovenbeentje. Als uw kindje ook een hepatitis B vaccinatie krijgt, wordt de pneumokokkenprik in een bovenarmpje gegeven. De prik op 24 maanden wordt thuis gegeven tijdens het laatste huisbezoek.

Tenslotte hopen wij bij een groep van ongeveer 190 kinderen een beetje bloed te mogen afnemen om vast te stellen hoe hoog de afweerstoffen zijn na inenting als ze 12 en 24 maanden oud zijnen. Hiervoor vragen wij u apart toestemming. U kunt dus ook aan het onderzoek meedoen als u alleen toestemming geeft voor het afnemen van neuswatjes.

Dagboekje na inenting

Prevenar® is een veilig vaccin dat al aan vele miljoenen baby's in de Verenigde Staten is gegeven, ook tegelijk met een DaKTP-Hib inenting. Toch vragen wij u om in het MINOES onderzoek na elke prik op 2, 4 en 11 maanden gedurende 7 dagen een dagboekje bij te houden om de eventuele bijwerkingen te noteren. Bovendien vragen we u gedurende 7 dagen na de prik de temperatuur van uw baby één keer per dag rectaal (via het poepgaatje) te meten en te noteren. Dit kost u elke dag 5-10 minuten tijd.

Wat vragen wij u nu te doen?

Indien u belangstelling heeft voor het onderzoek wilt u dan zo spoedig mogelijk, maar uiterlijk binnen twee weken, de portvrije antwoordkaart invullen en aan ons opsturen. Aan het onderzoek kunnen ongeveer 1000 gezinnen meedoen. Uw kindje kan de pneumokokkenvaccinatie krijgen voordat het landelijk wordt ingevoerd. In het geval u niet wilt meedoen, vragen wij u toch de antwoordkaart op te sturen (zonder telefoonnummer).

Ook als u al toestemming heeft gegeven voor het onderzoek staat het u altijd vrij, zonder opgaaf van redenen, deze toestemming weer in te trekken. Als u nog vragen heeft over het onderzoek kunt u altijd contact opnemen met een onderzoeksmedewerk(st)er (maandag t/m vrijdag van 9:00 tot 12:00 uur op telefoonnummer: 023-890 9070).

Wij hopen van harte dat u mee zult doen aan het MINOES onderzoek**.** Zo kunnen wij voor de kinderen het aantal prikken in het Rijksvaccinatieprogramma zo laag mogelijk houden en toch de kinderen goed beschermen tegen infectieziekten.

Met hartelijke groet,

Namens alle MINOES medewerkers

Mevr. Drs. E.J.M. van Gils Dr. R.H. Veenhoven Mevr. Prof. Dr. E.A.M. Sanders

Onderzoeksgroep

Lieke Sanders (Prof. dr.)	Kinderarts-immunoloog	Wilhelmina Kinderziekenhuis/UMC Utrecht
Reinier Veenhoven (Dr.)	Kinderarts	Spaarne Ziekenhuis Hoofddorp
Eelko Hak (Dr.)	Epidemioloog	Julius Centrum voor Huisartsgeneeskunde en Patientgebonden Onderzoek, UMC Utrecht
Elske van Gils (Drs.)	Arts-onderzoeker	Wilhelmina Kinderziekenhuis/UMC Utrecht
Arie van der Ende (Dr.)	Microbioloog	Nationaal Referentie Laboratorium voor Bacteriële Meningitis, Amsterdam
Ed IJzerman (Dr.)	Microbioloog	Stichting Streeklaboratorium voor de Volksgezondheid Kennemerland, Haarlem
Ger Rijkers (Dr. Ir.)	Medisch immunoloog	Laboratorium immunologie WKZ/UMC Utrecht

Onderzoek MINOES

Dit onderzoek is een samenwerkingsproject van het Universitair Medisch Centrum/Wilhelmina Kinderziekenhuis en het Julius Centrum in Utrecht, de afdeling kindergeneeskunde van het Spaarne Ziekenhuis Hoofddorp met subsidie van het ministerie van Volksgezondheid, Welzijn en Sport, vertegenwoordigd door het Nederlands Vaccin Instituut in Bilthoven. Aan dit onderzoek doen mee de Stichting Streeklaboratorium voor de Volksgezondheid Kennemerland te Haarlem, de Stichting Entadministratie Utrecht & Noord-Holland, de Stichting Regionale Entadministratie Zuid-Holland en de afdelingen Jeugdgezondheidszorg (0-4 jaar) van de Amstelring, de Thuiszorg IJmond, de Thuiszorg Zuid-Kennemerland, de Stichting Thuiszorg Groot Rijnland, Valent RDB en Florence.

Antwoordkaart MINOES

De ouders/verzorgers van …….,

Geboren … (dag)….(maand) 200.. (jaar)

☐ **Ja,** wij willen graag meer informatie over MINOES

en kunnen worden gebeld

Ons telefoonnummer is ----------

Een eventueel tweede nummer is -----------

☐ **Nee, wij hebben geen belangstelling voor het onderzoek,**

Wij zouden het fijn vinden als u eventueel de reden om niet aan het onderzoek mee te doen zou willen opschrijven. Dit geeft ons inzicht in uw motivatie

Wij doen niet mee omdat ....

Andere zijde kaart

Adres retour antwoordkaart naar adres MINOES
portvrij

Bijlage 2 Informatiebrief en informed consent formulier

Geachte ouders, verzorgers

Hartelijk dank voor uw aanmelding voor ons onderzoek MINOES. Zoals u in de informatiefolder heeft kunnen lezen, onderzoeken wij of in de toekomst binnen het Rijksvaccinatieprogramma met twee pneumokokkeninentingen in plaats van drie kan worden volstaan. Dit doen we door te vergelijken hoe vaak pneumokokken in de neus van uw baby en andere gezinsleden voorkomen na twee en drie keer prikken.

In deze informatiebrief leggen we de gang van zaken van het onderzoek nogmaals uit. Als bijlagen vindt u de toestemmingsformulieren voor het meedoen aan het onderzoek (verwijderd: ‘en een vragenlijst over de gezondheid en de omgeving van de baby’). We vragen u dit alles rustig door te lezen. Als u vragen heeft kunt u telefonisch contact met ons opnemen (maandag t/m vrijdag van 9:00 tot 12:00 uur op telefoonnummer: 023-890 9070). Ook zullen we bij het eerste huisbezoek voor het MINOES onderzoek (voordat uw baby 2 maanden oud is) nogmaals het onderzoek en het toestemmingsformulier (verwijderd: ‘en de vragenlijst’) met u doornemen.

Het eerste huisbezoek

Als u tijdens het huisbezoek besluit deel te nemen aan het onderzoek en uw baby gezond genoeg is om mee te doen, zullen u (beide ouders of wettelijk vertegenwoordigers) en de onderzoeksmedewerker de toestemmingsformulieren ondertekenen. Vervolgens wordt een eerste vragenlijst over de gezondheid en de omgeving van de baby met u doorgenomen en zullen wij ook het eerste neuswatje bij uw baby afnemen.

Daarna wordt door loting uw baby in één van de drie groepen ingedeeld.

1. Een pneumokokkenprik op de leeftijd van 2, 4 en 11 maanden
2. Een pneumokokkenprik op de leeftijd van 2 en 4 maanden
3. Een pneumokokkenprik aan het einde van het onderzoek op de leeftijd van 24 maanden

Noch u, noch wij kunnen zelf kiezen voor een groep.

Wij geven u vervolgens een brief om mee te nemen naar het consultatiebureau waarin staat welke inentingen uw kindje zal krijgen. De arts of verpleegkundige op het consultatiebureau zal de prikken geven. Alleen de prik op de leeftijd van 24 maanden (groep 3) geven wij zelf tijdens het laatste huisbezoek.

Wat zijn de bijwerkingen van de pneumokokkenprik Prevenar®?

Prevenar® bestaat uit bestanddelen van de buitenkant van 7 typen pneumokokken. De afweerstoffen die uw kindje maakt tegen deze bestanddelen beschermen hem/haar tegen hersenvliesontsteking en infecties van het bloed veroorzaakt door deze pneumokokken. De pneumokokkenprik bevat maar kleine stukjes van de buitenkant van de bacterie en dus niet de hele bacterie. Je kunt van de prik dus geen infectie krijgen en daardoor ziek worden. Wel activeert de pneumokokkenprik, net zoals de DaKTP-Hib vaccinatie, het afweersysteem van uw kindje. Daardoor kunnen bijwerkingen ontstaan zoals koorts van 38˚C tot 39˚C, of prikkelbaarheid (huilen), slaperigheid of juist onrustig slapen. De baby kan even wat last van verminderde eetlust, spugen of diarree hebben. Zelden (minder dan bij 1:1000 kinderen) treden stuipen of koortsstuipen op. Eveneens zelden treden allergische of overgevoeligheidsreacties op als uitslag/bultjes (netelroos) of jeuk op de plaats waar geprikt is of in het gezichtje (rode huidverkleuring om de oren), zwelling van de lippen, het gezichtje, om de ogen of in de neus, moeilijkheden met ademhalen of slikken, bloeddrukdaling met instorten/ shockachtig zijn.
De pneumokokkenprik wordt net als de DKTP-Hib prik in een beenspier toegediend, maar dan in het andere beentje. Als uw baby ook tegelijkertijd een hepatitis B vaccinatie moet krijgen, geven we de pneumokokkeninenting in een bovenarmpje. Op de prikplaats kan roodheid, pijn, zwelling of een wat vast aanvoelende plek ontstaan. Soms heeft uw baby even wat verminderde eetlust, spugen of diarree. Deze mogelijke bijwerkingen gaan vanzelf over binnen 24 tot 72 uur na de inenting. U kunt net als voor de DaKTP-Hib prik eventueel van tevoren paracetamol als kinderzetpil geven. De paracetamol kinderzetpil adviseren wij ook als uw kindje bekend is met koortsstuipen of epilepsie.

Dagboekje na inenting

Prevenar® is een veilig vaccin dat al aan vele miljoenen baby's in de Verenigde Staten is gegeven, ook tegelijk met een DaKTP-Hib inenting. Toch vragen wij u om in het MINOES onderzoek na elke prik op 2, 4 en 11 maanden gedurende 7 dagen een dagboekje bij te houden om de eventuele bijwerkingen te noteren. Bovendien vragen we u gedurende 7 dagen na de prik de temperatuur van uw baby één keer per dag rectaal (via het poepgaatje) te meten en te noteren. Dit betekent dat we aan alle ouders vragen gedurende 1 week het dagboekje bij te houden, ook als uw kindje alleen de DaKTP-Hib vaccinatie heeft gehad. Op deze wijze kunnen wij de bijwerkingen van Prevenar® vergelijken met die van de DaKTP-Hib inenting.

Huisbezoeken

Vervolgens komen wij 4 keer bij u thuis, zodra uw baby 6, 12, 18 en 24 maanden oud is. Wij nemen dan weer een vragenlijst met u door en nemen een neuswatje af bij uw baby. Op de leeftijd van 12 en 24 maanden, nemen we ook zo'n watje (en vragenlijst) af één van de eventuele broertjes of zusjes. Bij één van de ouders nemen we dan ook zo'n neuswatje én een keelwatje (en vragenlijst) af.

Apart toestemming voor bloedafname

Wij willen ook graag bij 190 kinderen een beetje bloed afnemen (per keer 3 ml, dus totaal 6 ml) om vast te stellen hoe hoog de afweerstoffen zijn na inenting als de baby 12 en 24 maanden oud is.

Hiervoor vragen we u apart toestemming. U kunt ook dus ook aan het onderzoek meedoen als u alleen toestemming geeft voor het afnemen van de neuswatjes.

Gegevens huisarts en ziekenhuis

Wij vragen u toestemming te geven om gegevens over uw baby op te vragen bij de huisarts en het ziekenhuis als uw baby ziek is geweest. Wij kunnen hiermee vaststellen hoe goed de pneumokokkenprik beschermt tegen infecties van de luchtwegen.

Hoe wordt de huisarts geïnformeerd?

Als u besluit mee te doen, krijgt U een brief mee met informatie voor de huisarts en eventueel behandelend specialisten. We vragen u om als u een arts bezoekt met uw kindje, deze brief aan de arts te geven zodat hij/zij kan lezen dat uw kindje meedoet aan MINOES en weet welke vaccinaties er zijn gegeven.

Wat gebeurt er met de gegevens?

Aan alle baby's, ouders en broertjes/zusjes die aan MINOES meedoen wordt een unieke code gegeven. Alle onderzoeksgegevens worden door ons onder deze code opgeslagen en in overeenstemming met de wet gedurende 15 jaar bewaard voor de onderzoekers.Het materiaal wordt zolang bewaard om eventueel later nog nader onderzoek te kunnen doen naar bacteriën of afweerstoffen tegen het vaccin. Alle gegevens worden vertrouwelijk behandeld zodat in de verslaglegging en publicaties niets meer terug te voeren is op uw kind of uw gezin. Onderzoeksgegevens kunnen alleen ingezien worden door daartoe bevoegde en gekwalificeerde onderzoeksmedewerkers, door bevoegde medewerkers van het Julius Centrum in Utrecht, door leden van de medisch-ethische toetsingscommissie of door vertegenwoordigers van de subsidiegever (VWS) en door medewerkers van het Nederlands Vaccin Instituut en hiertoe bevoegde autoriteiten. Tijdens het onderzoek kunnen daartoe bevoegde medewerkers (verwijderd van het Julius Centrum) komen kijken om te zien of het onderzoek loopt zoals met u is afgesproken. De inentingen worden net als alle andere inentingen geregistreerd bij de entadministratie.

Waar kunt u terecht met vragen?

Aan alle medewerkers van het onderzoek kunt u op elk moment vragen stellen. De telefoonnummers staan vermeld op dit formulier.

Onafhankelijke arts?

Als u twijfelt over deelname kunt u een onafhankelijke arts raadplegen, die zelf niet bij het onderzoek betrokken is, maar wel deskundig op het gebied van dit onderzoek. Voor MINOES is dit Dr. Peter de Winter, kinderarts in het Spaarne Ziekenhuis Hoofddorp (tijdens kantooruren op telefoonnummer 023-8908900). Ook als u voor of tijdens de studie vragen heeft die u liever niet aan de onderzoeksarts stelt, kunt u contact opnemen met deze onafhankelijke arts.

Wel of niet meedoen?

Meedoen aan dit onderzoek is geheel vrijwillig En natuurlijk kunt u op elk moment besluiten niet meer met het onderzoek mee te doen, ook als u eerder toestemming heeft gegeven. U hoeft hiervoor geen reden op te geven. Het heeft geen enkel gevolg voor de behandeling van u en uw baby. De onderzoekers stellen het belang van het kind voorop. Als wij menen dat het beter voor uw kind is niet mee te doen of te stoppen met het onderzoek, of als bijvoorbeeld de onderzoeksafspraken niet kunnen worden nagekomen, kunnen wij de deelname beëindigen.

Toestemmingsformulier tekenen?

Voor de wet dient het toestemmingsformulier door allebei de ouders/wettelijk verzorgers te worden getekend. Ook een bevoegde onderzoeksmedewerk(st)er tekent het toestemmingsformulier. U krijgt hiervan een kopie. U geeft apart toestemming voor het onderzoek met de neuswatjes, voor eventuele bloedafname en voor een eventueel vervolgonderzoek aan het einde van de studie.

Wat is het voordeel van meedoen aan de studie?

De pneumokokkenprik wordt totdat deze in het Rijksvaccinatieprogramma wordt opgenomen niet vergoed. Binnen MINOES krijgen twee van de drie kinderen die meedoen gratis Prevenar® vóór de leeftijd van 6 maanden, de derde groep krijgt de inenting op de leeftijd van 24 maanden. Uw kindje kan daardoor beter beschermd zijn tegen ernstige pneumokokkeninfecties. Uiteraard wordt de gezondheid van alle kinderen in het onderzoek nauwkeurig gevolgd.

Wat zijn de nadelen van deelname aan dit onderzoek?

Bij uw baby wordt 5 keer een kweek van het achterste deel van de neus (de neus-keelholte) afgenomen met een dun buigbaar staafje met een watje aan het uiteinde. Dit watje wordt diep in de neus ingebracht door een getrainde onderzoeksmedewerk(st)er en direct weer uitgenomen. Dit duurt maar heel kort maar geeft even een vervelend gevoel. Zeer zelden ontstaat een kleine bloedneus (minder dan bij 1 op de 1000 kinderen). Ook bij één van de ouders en broertjes/zusjes wordt twee keer zo'n neuswatje afgenomen. Bij de ouder wordt twee keer ook een keelwatje afgenomen. Hierbij wordt met een wattenstaafje achter in de keel wat slijm afgenomen. Ook dit duurt maar heel kort maar geeft even een vervelend gevoel achterin de keel.

Als bij uw baby ook bloed wordt geprikt, gebeurt dit door een getrainde en ervaren arts. Beide malen wordt een kleine hoeveelheid (per keer 3 milliliter, totaal 6 milliliter) afgenomen, dit is zo weinig dat uw kind daar geen last van heeft. Wel kan het prikken wat pijnlijk zijn en soms ontstaat op de plaats van het prikken een kleine blauwe plek, die vanzelf binnen een paar dagen weer wegtrekt. Wij willen graag bloed prikken bij 30 kinderen uit de onderzoeksgroep die de vaccinatie pas krijgt op 24 maanden en bij 80 kinderen uit elk van de andere twee groepen.

Het bijhouden van de dagboekjes na de inentingen en het invullen van de vragenlijsten bij het huisbezoek kosten natuurlijk tijd. In totaal zijn er vijf huisbezoeken met vragenlijsten en neusuitstrijkjes. Elk bezoek zal een 30 tot 45 minuten in beslag nemen. Het invullen van het

dagboekje gedurende een week na de inentingen en het meten van de temperatuur bij uw kindje kost in totaal ongeveer 5 tot 10 minuten.

Wat gebeurt er als u niet deelneemt?

Uw kind krijgt op het consultatiebureau gewoon de zorg en de vaccinaties volgens het standaard Rijks vaccinatieprogramma. Persoonlijke gegevens worden niet aan de onderzoekers verstrekt.

Verzekering van het onderzoek en klachten

Tenslotte moeten de onderzoekers volgens de Wet Medisch-Wetenschappelijk Onderzoek met Mensen (WMO), verzekeringsbesluit 2003, een verzekering afsluiten bij elk onderzoek voor elke deelnemer, mocht er toch iets gebeuren met uw kindje (zie verzekeringsbijlage). Dit hebben wij ook gedaan. Indien er naar uw mening schade is ondervonden, omdat u denkt dat er fouten zijn gemaakt of uw kind schade ondervindt door deelname aan dit onderzoek, moet u dit zo snel mogelijk melden bij de verzekering. Bij klachten over het onderzoek kunt u zich wenden tot de onderzoeker of, als u dit wilt, bij het Bureau Patiëntenservice van het UMC Utrecht (tijdens kantooruren op telefoonnummer: 030-2508850)

Financiering van het onderzoek

De onderzoeksgroep van MINOES, een samenwerking tussen het Universitair Medisch Centrum/ Wilhelmina kinderziekenhuis, het Julius Centrum te Utrecht en het Spaarne Ziekenhuis te Hoofddorp, heeft subsidie van het Ministerie van Volksgezondheid, Welzijn & Sport gekregen om het onderzoek uit te voeren.

Tenslotte

Nogmaals hartelijk dank voor uw bereidheid mee te willen werken aan dit onderzoek. Het kan betekenen dat wij in de toekomst in het Rijksvaccinatieprogramma met minder prikken de kinderen toch goed kunnen beschermen.

Met hartelijke groet,

Namens alle MINOES medewerkers

Mevr. Drs. E.J.M. van Gils	Dr. R.H. Veenhoven	Mevr. Prof. Dr. E.A.M. Sanders
Arts-onderzoeker	Kinderarts	Kinderarts-immunoloog

Leeftijd baby	Onderzoek	Tijdsbeslag ouders
2-4 weken<br><br>Informatiekaart bij MINOES terugsturen	Ontvangst informatiefolder, terugsturen antwoordkaart<br><br>**Telefonisch contact**: toesturen informatie-brief en toestemmingsformulier. Na 7 dagen opnieuw telefonisch contact voor het maken van afspraak eerste huisbezoek vóór dat de baby 2 maanden oud is	Samen 40 minuten
4-7 weken	**Huisbezoek:** vragenlijst doornemen, toestemmingsformulier tekenen, eerste neuswatje bij de baby. Loten welke	30-45 minuten

	inentingen de baby gaat krijgen	
2 maanden	**Consultatiebureau**: afhankelijk van de groep waarin de baby is ingedeeld **Prevenar®** inenting tegelijk met DaKTP-Hib (en eventueel hepatitis B). **Dagboekje** gedurende een week bijhouden en gedurende die dagen de **temperatuur** meten.	Dagboekje invullen en temperatuur meten kost 5-10 minuten per dag
4 maanden	**Consultatiebureau:** afhankelijk van de groep waarin de baby is ingedeeld **Prevenar®** inenting tegelijk met DaKTP-Hib (en eventueel hepatitis B). **Dagboekje** gedurende een week bijhouden en **temperatuur** meten.	Dagboekje invullen en temperatuur meten kost 5-10 minuten per dag
6 maanden	**Huisbezoek:** vragenlijst doornemen en neuswatje bij de baby	30 minuten
11 maanden	**Consultatiebureau:** afhankelijk van de groep waarin de baby is ingedeeld **Prevenar®** inenting tegelijk met DaKTP-Hib (en eventueel hepatitis B). **Dagboekje** gedurende een week bijhouden en die dagen de temperatuur meten.	Dagboekje invullen en temperatuur meten kost 5-10 minuten per dag
12 maanden	**Huisbezoek:** vragenlijst en neuswatje bij de baby, een broertje of zusje en één van de ouders. Keelwatje bij één van de ouders.Bij een deel van de baby's een bloedprik	45 minuten
18 maanden	**Huisbezoek:** vragenlijst doornemen en neuswatje bij de baby	30 minuten
24 maanden	**Huisbezoek:** vragenlijst en neuswatje bij de baby, een broertje of zusje en één van de ouders. Keelwatje bij één van de ouders.Bij een deel van de baby's bloedprik.<br>**Afsluiten onderzoek** en Prevenar® inenting door arts als deze nog niet eerder gegeven zijn.	45 minuten

Verzekeringsbijlage

Voor de deelnemers aan dit onderzoek is een verzekering afgesloten. Deze verzekering dekt schade door dood of letsel die het gevolg is van deelname aan het onderzoek, en die zich gedurende de deelname aan het onderzoek openbaart, of binnen vier jaar na beëindiging van de deelname aan het onderzoek. De schade wordt geacht zich te hebben geopenbaard wanneer deze bij de verzekeraar is gemeld.

In geval van schade kunt u zich direct wenden tot de verzekeraar:

Naam:	Gerling Allgemeinde Versicherungs-AG
Adres:	Postbus 2636
	1000 CP Amsterdam
Telefoonnummer:	020 - 54 92 213
Contactpersoon:	mr. P. Oosterveen

De verzekering biedt een maximum dekking van € 450.000 per proefpersoon en € 3.500.000 voor het gehele onderzoek en € 5.000.000 per jaar voor alle onderzoeken van dezelfde opdrachtgever. De dekking van specifieke schades en kosten is verder tot bepaalde bedragen beperkt. Dit is opgenomen in het Besluit verplichte verzekering bij medisch-wetenschappelijk onderzoek met mensen. Informatie hierover kunt u vinden op de website van de Centrale Commissie Mensgebonden Onderzoek: www.ccmo.nl.

Voor deze verzekering gelden een aantal uitsluitingen. De verzekering dekt niet:

- schade waarvan op grond van de aard van het onderzoek zeker of nagenoeg zeker was dat deze zich zou voordoen;
- schade aan de gezondheid die ook zou zijn ontstaan indien u niet aan het onderzoek had deelgenomen;
- schade die het gevolg is van het niet of niet volledig nakomen van aanwijzingen of instructies;
- schade aan nakomelingen, als gevolg van een nadelige inwerking van het onderzoek op u of uw nakomeling;
- bij onderzoek naar bestaande behandelmethoden: schade die het gevolg is van één van deze behandelmethoden;
- bij onderzoek naar de behandeling van specifieke gezondheidsproblemen: schade die het gevolg is van het niet verbeteren of van het verslechteren van deze gezondheidsproblemen.

Toestemmingsformulier MINOES

INFORMED CONSENT

Studienummer _____________

(Datum verwijderd)

Informed consent betreffende het onderzoek MINOES:

Aan Dhr/Mevr ………………………………………………………………………………
Ouders/verzorgers van ………………………………………………………………………………
Geboortedatum ___ / ___ / ______

Zijn de achtergrond, doelstelling en de gang van zaken van het onderzoek met het pneumokokkenvaccin Prevenar® goed uitgelegd.

Het is ondergetekenden duidelijk dat:

hij/zij niet verplicht is met zijn/haar kind aan dit onderzoek deel te nemen

hij/zij op ieder willekeurig tijdstip en zonder opgaaf van redenen zijn/haar kind uit het onderzoek kan terugtrekken

de beslissing om al dan niet aan het onderzoek deel te nemen of om er voortijdig mee te stoppen niet van invloed is op de verdere behandeling van zijn/haar kind.

het ondertekenen van het informed consent formulier betekent dat gegevens van het kind betreffende bezoeken aan de huisarts of een specialist door bevoegde onderzoekers, leden van de medisch ethische commissie of andere bevoegden als medewerkers van het Julius Centrum die de onderzoeksprocedures controleren, kunnen worden ingezien

alle gegevens voortkomend uit het onderzoek geanonimiseerd worden verwerkt ten behoeve van het beantwoorden van de onderzoeksvragen en publicaties. Dat wil zeggen dat in publicaties of presentaties gegevens niet meer tot het kind of gezinsleden te herleiden zijn

Het is bovendien duidelijk dat

het gecodeerde kweekmateriaal 15 jaar wordt bewaard voor nadere analyses en wetenschappelijk onderzoek en daarna vernietigd

………………………	………………………	……………………………………………
Plaats	Datum	Naam en handtekening ouder/verzorger
………………………	………………………	……………………………………………
Plaats	Datum	Naam en handtekening ouder/verzorger
………………………	………………………	……………………………………………
Plaats	Datum	Naam en handtekening onderzoeksmedewerker

Toestemmingsformulier MINOES bloedprik

INFORMED CONSENT

Studienummer _____________

(Verwijderd Datum)

Informed consent betreffende het onderzoek MINOES:

Aan Dhr/Mevr ……………………………………………………………………….
Ouders/verzorgers van ……………………………………………………………………….
Geboortedatum ___ / ___ / ______

Mij zijn de achtergrond, doelstelling en de gang van zaken van het onderzoek met het pneumokokkenvaccin Prevenar® goed uitgelegd.

Ik heb (verwijderd wel)geen bezwaar dat behalve de kweken uit de neus ook 3 milliliter bloed wordt afgenomen op de leeftijd 1 en 2 jaar (totaal 6 ml) bij mijn kind om het bloed te controleren op afweerstoffen tegen pneumokokken

Het is bovendien duidelijk dat als ik toestemming heb gegeven voor bloedafname, dit materiaal uitsluitend wordt bekeken in relatie tot afweer tegen pneumokokken en dat het na 15 jaar wordt vernietigd

………………………	………………………	………………………………………………
Plaats	Datum	Naam en handtekening ouder/verzorger

………………………	………………………	………………………………………………
Plaats	Datum	Naam en andtekening ouder/verzorger

………………………	………………………	………………………………………………
Plaats	Datum	Naam en handtekening onderzoeksmedewerker

Toestemmingsformulier MINOES vervolg

INFORMED CONSENT
Studienummer …………
(Verwijderd datum)

Informed consent betreffende het onderzoek MINOES:

Aan Dhr/Mevr ………………………………………………………….
Ouders/verzorgers van ………………………………………………………….
Geboortedatum ………………………………………………………….

Mij zijn de achtergrond, doelstelling en de gang van zaken van het onderzoek met het pneumokokken vaccin Prevenar® goed uitgelegd.

Ik heb (verwijderd wel/) geen bezwaar om over twee jaar (verwijderd 'opnieuw') te worden benaderd voor vervolgonderzoek. Ik geef over twee jaar daar al dan niet opnieuw toestemming voor zodra duidelijk is wat dit onderzoek dan inhoudt.

………………………	………………………	………………………………………………
Plaats	Datum	Naam en handtekening ouder/verzorger

………………………	………………………	………………………………………………
Plaats	Datum	Naam en andtekening ouder/verzorger

………………………	………………………	………………………………………………
Plaats	Datum	Naam en<br>handtekening onderzoeksmedewerke
