# Supplementary material for: Effect of Seven-Valent Pneumococcal Conjugate Vaccine on Staphylococcus aureus Colonisation in a Randomised Controlled Trial
Source: PLoS One. 2011 Jun 10;6(6):e20229. doi: 10.1371/journal.pone.0020229 (PMC3112202; doi:10.1371/journal.pone.0020229)
Supplement: Summary Protocol S1 — Summary trial protocol. (PDF) [file pone.0020229.s003.pdf]

SUMMARY STUDY PROTOCOL

Effect of two and three Prevenar® vaccinations in the NIP on pneumococcal nasopharyngeal carriage, transmission and herd immunity: a randomized controlled study.

Short title: Influence of Prevenar on Nasopharyngeal Carriage and Herd Immunity

Original language protocol: Dutch

Financial support

Dutch Ministry of Health

Medical Ethical Approval

The study protocol was approved by an acknowledged Medical Research Ethics Committee: STEG (Dutch: Stichting Therapeutische Evaluatie Geneesmiddelen, www.stegmetc.org), responsible for reviewing medical research involving human subjects in the Netherlands (protocol reference: STEG R05 008).

Trial Registration

ISRCTN: 25571720 (date created: Dec 20th, 2005; date requested: Oct18th, 2005)

ClinicalTrials.gov Identifier: NCT00189020

Dutch Trial Register: NTR155

Purpose

A 2+1-dose and even a 2-dose instead of the licensed 3+1-dose schedule with pneumococcal conjugate vaccine (PCV-7) are presumed to protect children against invasive pneumococcal disease like meningitis, at least on the short term till 18-24 months of age. The hypothesis in this study is that a 2- and 2+1-dose schedule will protect against invasive pneumococcal disease (IPD) but may have less effect on pneumococcal nasopharyngeal carriage in infants, and consequently affect pneumococcal transmission and herd-immunity. Reduced doses may have less effect on replacement by non-vaccine serotypes and other potentially pathogenic bacteria in the nasopharynx. Furthermore, antibody development and memory may benefit from carriage of vaccine type *S. pneumoniae.*

Study characteristics

Study type: Interventional

Study Design: Randomized, Parallel Assignment, Superiority Study

Enrolment: 1003 healthy newborns (and family members)

Study Start Date: June 2005

Study Completion Date: March 2008

Outcome Measures

Primary Outcome Measures:

- Nasopharyngeal VT pneumococcal colonization in infants in the second year of life.

Secondary Outcome Measures:

- Nasopharyngeal NVT pneumococcal colonization in the second year of life.
- Nasopharyngeal VT pneumococcal colonization in family members (parent, sib) at index child's age of 12 and 24 months.
- Nasopharyngeal NVT pneumococcal colonization in family members at index child's age of 12 and 24 months.
- Nasopharyngeal colonization of *M. catarrhalis*, *H. influenzae*, *S. aureus* in infants in the second year of life.
- Nasopharyngeal colonization of *M. catarrhalis*, *H. influenzae*, *S. aureus* in family members at index child's age of 12 and 24 months.
- Anti-pneumococcal antibody levels in infants at 12 and 24 months of age.

Detailed Description

A 2+1-dose (at age 2, 4 and 11 months) and even a 2-dose (age 2 and 4 months) vaccination schedule with 7-valent pneumococcal conjugate vaccine Prevenar in infants are presumed to provide about 90% protection against invasive pneumococcal disease (IPD) for vaccine-type pneumococci, at least until 18-24 months of age. Licensure of the vaccine however is based on studies with 3 vaccinations before 6 months and a booster vaccination in 6 months later (3+1-schedule). A 3+1-dose PCV-7 schedule results in a 50% reduction of nasopharyngeal (NP) pneumococcal colonization of those serotypes included in the vaccine. In the USA, substantial herd-immunity has been reported for unvaccinated individuals, attributed to the reduced carriage of pneumococcal vaccine-types in vaccinated infants, who are a primary source of spread for pneumococci in the community. Cost-effectiveness of PCV-7 implementation in national infant vaccination programs (NIP) has much improved by incorporating these herd effects. The overall pneumococcal colonization in infants however was not reduced after 4-dose PCV-7 schedule due to a similar increase in carriage of pneumococcal non-vaccine serotypes (so-called replacement). An increase of replacing non-vaccine-type pneumococci in diseases like otitis media and invasive pneumococcal disease has been reported. A 2+1- and even a 2-dose PCV-7 schedule are presumed to offer sufficient protection against IPD, but may affect carriage reduction of vaccine-type pneumococci and may therefore on the one hand induce less herd effect for vaccine-type IPD and respiratory tract infections, but on the other hand may also cause less replacement with non-vaccine-type pneumococci and other potentially pathogenic bacteria in the nasopharynx. Also, ongoing natural boosting of immunity by frequent colonization of vaccine serotypes may result in long-term persistence of protective anti-pneumococcal serum antibody levels both in infants and the community.

Aims

The primary aim of the current study is to compare the effect of a 2-dose (at ages 2 and 4 months) and a 2+1-dose PCV-7 schedule (at 2, 4 and 11 months) with unvaccinated controls on nasopharyngeal vaccine-serotype pneumococcal carriage The secondary aim is to determine the effect of a reduced doses schedule on NVT carriage (replacement) in vaccinated infants, nasopharyngeal colonization in parents and sibs, serum anti-pneumococcal antibody levels at the age of 12 and 24 months. Furthermore, to determine nasopharyngeal colonizing pneumococci in unvaccinated infants in the Netherlands before implementation of Prevenar in the NIP, to evaluate the effect of PCV-7 on other colonizing bacteria like *H. influenzae, M. catarrhalis* and *S. aureus*, and the relation between colonizing pneumococci and serotypes causing IPD in the Netherlands.

Methods

Sample size calculation

The needed sample size per study-group (330) was calculated on the assumption of a peak VT carriage rate of 35% in children at the age of 12 months based on previous experience and an estimated 33% reduction of VT-carriage in the 2-dose and 2+1-dose group respectively, with 80% power at a 5% significance level and taking a 10% drop out rate into account. Statistical analyses follow the intention-to-treat principle.

Randomisation

Infants are randomly assigned via a computer randomisation interface to one of the 3 study groups:

1. Prevenar at 2 and 4 months
2. Prevenar at 2, 4 and 11 months
3. Prevenar at 24 months (controls)

The children will be followed until 2 years of age with nasopharyngeal swabs for bacterial culture (*S. pneumoniae, H. influenzae, M. catarrhalis, S. aureus*, haemolytic streptococci group A, B, C and G, *B. (para)pertussis*) before the first vaccination, at 6, 12, 18 and 24 months of age. One sibling and one parent/caregiver will be swabbed when the infant is 12 and 24 months. Blood for antibody determination (double adsorption ELISA) will be obtained from 80 children of groups 1 and 2, and from 30 children in the control group. Questionnaires on demographic factors, risk factors and respiratory tract infections (RTI) and antibiotic prescription for RTI will be obtained.

Eligibility Criteria

Ages eligible for study: <12 weeks of age

Genders eligible for study: Both

Inclusion Criteria:

- Newborn infants eligible for participation in the national infant vaccination program in the Netherlands

Exclusion Criteria:

Exclusion from the national immunization program because of the presence of:

- A medical condition requiring treatment that can interfere with the effect of vaccinations
- Known or suspected allergy to components of the pneumococcal conjugate vaccine
- Known or suspected immunodeficiency disease

- Previous treatment with plasma or immunoglobulin
- Previous vaccinations other than hepatitis B vaccinations
- Coagulation disorders

Sponsors and Collaborators

Principal Investigator: Professor Elisabeth A. M. Sanders, MD, PhD, University Medical Center Utrecht

Project Coördinator: Reinier H. Veenhoven, MD, PhD, Spaarne Hospital
